# Microplastics and intI1 antibiotic resistance-associated marker in rural water systems: an exploratory GIS-based environmental assessment

**DOI:** 10.3389/fmicb.2026.1912851

**Published:** 2026-09-18

**Authors:** Sneha Gautam, Bairi Levi Rakshith, Jerlin Joy, Anu Jacob, D. Subith, Ashlin A, Lazarus Godson Asirvatham, Dennis Jose, Baehyun Min

**Affiliations:** 1 Division of Civil Engineering, Karunya Institute of Technology and Sciences, Deemed University, Coimbatore, Tamil Nadu, India; 2 Department of Climate and Energy Systems Engineering, Ewha Womans University, Seoul, Republic of Korea; 3 Division of Mechanical Engineering, Karunya Institute of Technology and Sciences, Coimbatore, Tamil Nadu, India; 4 Centre for Research in Material Science and Thermal Management (CRMS and TM), Karunya Institute of Technology and Sciences, Coimbatore, Tamil Nadu, India; 5 Division of Biotechnology, Karunya Institute of Technology and Sciences, Coimbatore, Tamil Nadu, India

**Keywords:** environmental contamination, GIS-based spatial analysis, *intI1* antibiotic-resistance-associated marker, microplastics, rural water systems

## Abstract

Microplastics and antibiotic resistance markers are two emerging contaminants, especially in rural areas with no central waste treatment. This study explored the spatial distribution and co-occurrence of microplastics and the *intI1* antibiotic-resistance-associated marker in the rural water bodies in Madavarayapuram, Tamil Nadu, India using a GIS-based exploratory framework implementing the SDG 3, SDG 6, and SDG 13. Seventeen drinking, tap, wastewater and river water samples were collected and analyzed by Fourier Transform Infrared; polyolefins were discovered, predominantly polypropylene in drinking water, despite the latter revealing zero turbidity. The polyolefin fractions varied by site, with tap water containing 95.73% relative polyolefin fraction, the wastewater at 86.79%, and the wastewater having substantial turbidity at 14.3 NTU, suggests that there is variation in relative polymer fractions and turbidity of the screened water based on sampling site. Samples were gathered at many sites along the river, although few samples were taken, so spatial patterns are difficult to analyze. However, there was an important difference between water types in turbidity (Kruskal-Wallis, *p* = 0.043) but not in polymer composition (*p* = 0.748). The presence of culturable bacterial populations in each of the samples was confirmed by microbial analysis, and Presence of the *intI1* antibiotic-resistance-associated marker (approximately 280 bp) can be detected qualitatively by PCR. However, detection of the *intI1* marker by PCR does not guarantee that antibiotic-resistant bacteria are present. The method does not identify antibiotic resistance. The detection of microplastics and the antibiotic resistance gene, *intI1*, in all environmental water matrices that were tested suggests an associated co-occurrence, however it does not suggest or prove that there is a direct correlation between microplastics and the occurrence of antibiotic resistance transmission. These findings highlight the limitations of conventional monitoring approaches and the need for integrated and thorough rural water supply systems to ensure safety.

## 1 Introduction

Plastic pollution constitutes one of the most popular and high-profile environmental issues of the twenty-first century, with over 400 million metric tons of plastic produced worldwide each year (Nayanathara Thathsarani Pilapitiya and Ratnayake, 2024). Plastic production continues to increase due to the popularity of single-use plastic packaging, plastic consumer goods, plastic agriculture and plastic infrastructure (Stanley et al., 2025). Much of the plastic entering the environment can be attributed to mismanaged waste, macroplastic debris fragmentation and breakdown of synthetic polymer materials (Kumar et al., 2025). Weathering by the effects of time, ultraviolet radiation and mechanical abrasion reduces macroplastic debris into microparticles of plastic, termed microplastics, defined as any plastic debris < 5 mm in size (Rakshith et al., 2025a). Microplastics have been widely reported in marine, freshwater, terrestrial and atmospheric environments, including in drinking water supplies, raising concerns about their persistence in the environment and potential impacts on ecosystems and human health (Gautam et al., 2025). In addition to being passive environmental pollutants, microplastics are increasingly acknowledged to represent biologically active surfaces for the colonization by microbes and biofilms in marine and freshwater environments (Mousazadehgavan, 2026). The hydrophobic polymer surfaces and the high surface area to volume ratio of microplastics ease their exposure to microbial colonization and the establishment of complex microbial assemblages, or the plastisphere (Yadav et al., 2026). Biofilm-associated microplastics can be colonized by microbial communities of opportunistic pathogens, environmental bacteria and ease horizontal gene transfer between microbes from different species (Rakshith et al., 2025b). Microplastics are pollutants and transient microhabitats with the potential to change community composition and structure and may enable microbes to transfer between environmental compartments (Jolaosho et al., 2025). However, many recently published studies by Zhang et al. (2026), Luo et al. (2023), Jangid et al. (2026), and Ahmad et al. (2024), and Goswami et al. (2025). have found that microplastics may also contribute to disseminating ARGs in the environment. The World Health Organization has described antibiotic resistance as one of the global public health threats and is defined by two key phenomena: the emergence of resistant microorganisms and the dissemination of resistance determinants in environmental reservoirs (World Health Organization, 2023). Aquatic environments are being recognized as key reservoirs and vectors of antibiotic resistance, due to continuous anthropogenic introduction of domestic sewage, fertilizer runoff, pharmaceutical concentrations in many types of waters, and poor waste stream management (Meradji et al., 2025).

Additionally, the surfaces of microplastics may promote the development of biofilm, which could help ease the accumulation of microbes and horizontal gene transfer, and consequently, the environmental dissemination of resistance genes (Zhang et al., 2025). Wide acceptance for the class 1 integrase Gene, *intI1*, as a molecular marker of anthropogenic pollution of environments by antibiotic-resistant bacteria has led scientists to consider it a key indicator of human impact on the environment and the presence of mobile genetic elements (Haenelt et al., 2023). Research into how microplastics could be helping the spread of microbes and antibiotic resistance has raised interest, however to date, most of this work has been carried out in the marine environment or urban wastewater systems (Siddique et al., 2025). Though less well studied, rural water systems are also vulnerable, especially because in many rural communities, drinking water sources, wastewater systems, agricultural run-off pathways, and unregulated open dumping sites are in close proximity to each other in a small geographic area (Wadumestrige Dona et al., 2025). The absence of municipal waste management practices and advanced sewage treatment infrastructure increases the probability of contamination with plastic waste, microbial contaminants, and antibiotic resistance genes (Hongjin et al., 2025). Rural and non-urban areas have been identified as important but neglected reservoirs for the persistence and dissemination of antibiotic resistance (Singh and Kim, 2025). To study the diffusion of microplastic contamination and the associated microbial hazard in such environments environmental monitoring and spatial analysis are needed.

Geographic Information System (GIS) technologies can be a useful mechanism for analyzing the spatial relationships between environmental components, potential spatial pattern and clustering, and potential transportation routes across complex geographic areas (Dong et al., 2025). The use of GIS tools to integrate spatial data with environmental sampling information (e.g., land use, hydrology, and terrain information) can be valuable in illustrating the processes by which contaminants move through the environment, and propagating environmental risk (Alagulakshmi et al., 2025). The study intended to investigate the occurrence and co-occurrence of microplastics, bacteria, and the *intI1* antibiotic-resistance-associated marker in rural water bodies. A GIS-based sampling scheme was implemented in a representative rural area to assess the occurrence and spatial distribution of microplastics in four functional microhabitats: drinking water sources, water storage facilities, water bodies impacted by waste, and drainage or runoff convergence points. Thus, the goal was not to predict local spatial variability on a regional scale, but rather to conduct a village-scale GIS study of environmental heterogeneity.

Microplastics were isolated and characterized using microscopy and spectroscopy techniques. Polymer types were qualitatively analyzed. Bacteria were preliminarily characterized using culture techniques and Gram-staining tests, with DNA extraction and polymerase chain reaction (PCR) amplification of the *intI1* antibiotic-resistance-associated marker. Spatial mapping and interpolation analyzes were used to identify exploratory geographical patterns. Spatial autocorrelation (Moran’s I), hotspot analysis, and interpolation validation were used to assess statistical confidence in exploratory patterns and their uncertainty. This study provides an integrated framework for the environmental surveillance of emergent contamination scenarios at the village level through the characterization of microplastics, microorganisms, and geospatial analysis of the environment. These results provide preliminary evidence of the co-occurrence of microplastics and the *intI1* antibiotic-resistance-associated marker in the environment, shedding light on the potential for microplastic-associated biofilm formation, horizontal gene transfer, and consequent dissemination of antibiotic resistance. These findings have important implications for monitoring and mitigation strategies for rural drinking water sources, and for protecting overall environmental and public health.

## 2 Materials and methods

### 2.1 Study area and sampling design

The study area lies in Madavarayapuram village of Coimbatore district, Tamil Nadu, India in the Noyyal River Basin (10.93–10.95 ° N Latitude, 76.73–76.75 ° E Longitude) as given in Figure 1. Present study area is an example of a rural settlement consisting of clusters of houses, agricultural fields, drainage channels, and small water bodies in a small area. These environmental factors influence the accumulation and transport of microplastics and associated microbial pollutants through surface runoff, direct discharge of wastewater, and hydrological connectivity. The study area was defined using satellite imagery and administrative boundary data in a geographic information system (GIS). Spatial analysis and mapping of these contaminant transport pathways were conducted in ArcGIS Pro. Spatial datasets used to analyze these pathways included satellite imagery basemaps, Digital Elevation Models (DEMs), Land Use/Land Cover (LULC) datasets, hydrology datasets (rivers, drainage network, sub-basins), and the georeferenced sampling sites, which helped analyze the environmental variables affecting contaminant transport pathways. Figure 2 presents a GIS-based spatial data integration framework for environmental interpretation.

**FIGURE 1:**
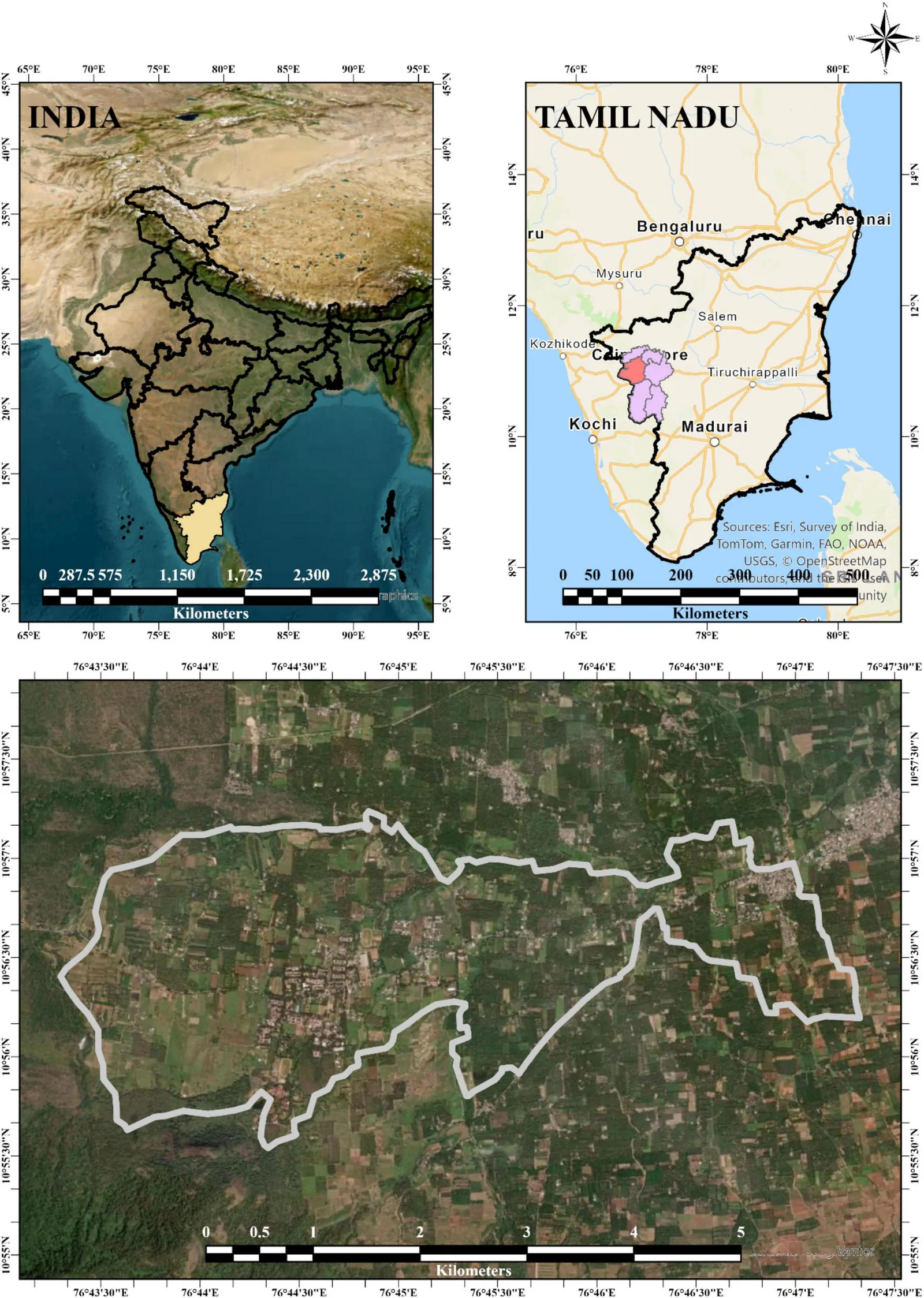
Location map of the sampling sites within the Madavarayapuram study area, Coimbatore district, Tamil Nadu, India. The spatial layout of the study area was prepared using ArcGIS Pro based on georeferenced sampling locations and supporting spatial datasets.

**FIGURE 2:**
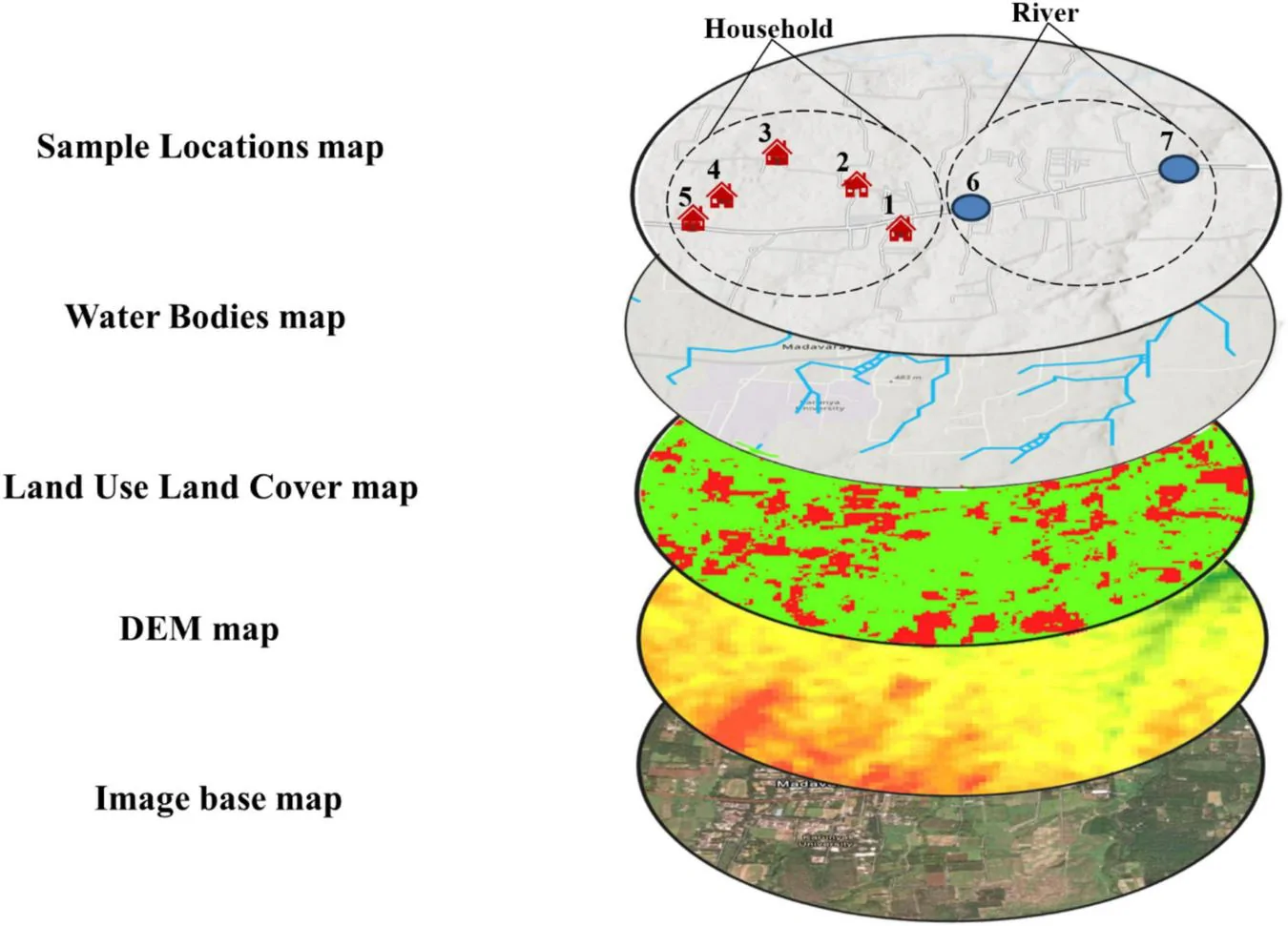
Integration of spatial datasets, including satellite imagery basemap, Digital Elevation Model (DEM), Land Use/Land Cover (LULC), water bodies, and sampling locations, used for GIS-based interpretation of the study area. The map was generated using ArcGIS Pro.

A GIS-based sampling approach was applied to characterize the spatial heterogeneity of the village water system. Four functional microenvironment zones were delineated on the basis of water-use patterns and exposure to plastic waste and anthropogenic inputs. Water zones identified were S1 (drinking water source zones), where hand pumps, borewells and open wells were used for public water supply; S2 (water storage zones), which included household water storage tanks and public taps; S3 (waste influenced water zones), where water was found in stagnant water bodies or areas near plastic dumping grounds; and S4 (drainage or runoff convergence zones), where water was found in surface runoff and graywater. 17 water samples were collected, five each from S1, S2, and S3 and two from S4. All samples were spatially referenced using a Global Positioning System (GPS) for mapping and interpolation. The sampling was not designed to be a repeated seasonal sampling program; instead, the goal was to characterize spatial variability of the different water types in the study area. Overall, seasonal and interannual variability were beyond the scope of this study. All field sampling was conducted under contamination control protocols to minimize the introduction of microplastics from external sources. Glass sampling bottles were washed with laboratory detergent, rinsed with distilled water, filtered with deionized water, and covered with aluminum foil. Study personnel wore cotton laboratory coats and powder-free nitrile gloves when collecting samples. Field blanks of filtered deionized water were left open and exposed at each of the sampling sites and were also processed with the environmental samples to monitor for contamination during sampling and processing.

### 2.2 Microplastic isolation and polymer identification

Microplastic particles in the water collected from a selected site were separated from the water by filtering the sampled water. Water samples were agitated prior to analysis to evenly disperse particles in the water. The dispersed, known volumes of water were filtered through polycarbonate membrane filters with 0.05 m pores under laboratory conditions to collect the suspended material. Membranes with trapped material were put on cleaned glass Petri dishes under open laboratory benches with low contamination levels. Membranes were left to dry. The dried filters were then analyzed using a stereomicroscope. Microplastic particles were identified based on characteristics such as color, surface structure and shape. These particles were then sorted into common microplastic morphologies, such as microplastic fragments, fibers and microplastic films. Qualitative morphological observations were the primary focus. Methodical in-depth measurements of particle size, morphology, and color have not always been done. The methodology of relying on visual inspection was adopted as a first step in the screening process before polymer confirmation. To identify the chemical composition of the microplastic particles identified visually, representative microplastic samples of the particles within each sampling station were analyzed with Fourier Transform Infrared (FTIR) spectroscopy using the Shimadzu IRPrestige-21 FTIR spectrophotometer (Shimadzu Corporation, Japan). The representative microplastic particles were carefully scraped off the filters, mixed with dry potassium bromide (KBr) powder, and ground together using a mortar and pestle. The mixture was then compressed in a hydraulic press into a KBr tablet that was translucent. Infrared spectroscopy was then performed with a blank KBr tablet used for calibration. The spectrum of the KBr pellet was recorded over the range 4,000–400 cm^–1^ with a spectral resolution of 4 cm^–1^ over 20 scans. The sample pellets were prepared and analyzed under the same instrumental parameters. The spectra were baseline corrected, normalized, and smoothed. The polymers were identified by matching the positions of the characteristic absorption bands to reference spectra from standard polymer libraries. Polymers were only identified when the major diagnostic absorption features of the reference spectra matched the sample spectra. Numerical library matching scores were not generated on the analytical laboratory results and were not recalculated retrospectively. The identification of PE and PP was done via the characteristic C-H stretching and CH_3_ bending bonded group vibrations in the 2,920–2,850 and 1,380–1,390 cm^–1^ range respectively. The FTIR investigation focused on the polyolefin bands of PE and PP and other polymer classes shown in the table were not explored without bias. This means that their presence cannot be ruled out. The FTIR software version used to obtain the spectra was not specified in the analytical laboratory report. The spectroscopic analysis found characteristics of polyethylene and polypropylene polyolefin contamination in the analyzed samples, which suggested that packaging materials and the use of household and agricultural plastic products were probable anthropogenic sources of microplastic contamination in the rural waterway. Microplastic recovery efficiencies from spiked reference particles were not measured in the present study, therefore, the percentages of polymer types represent the relative polymer composition of analyzed particles and not the absolute microplastic abundance or recovery.

### 2.3 Microbial analysis

Bacterial analyzes were conducted to preliminary characterize the types of bacteria present in the water samples through microbial culturing, isolation of colonies, and microscopy via Gram staining. The nutrient agar medium used in this experiment was prepared by mixing the nutrient agar powder (4.8 g) in 400 mL of distilled water and heating it with constant stirring until it was dissolved out. Then, it was autoclaved at 121 °C and 15 psi. The medium was allowed to cool to about 45–50 °C before being poured aseptically under laminar airflow conditions into sterile Petri plates. After the plates were allowed to strengthen, 100 μL of each water sample was aseptically inoculated onto the already prepared nutrient agar plates. The plates were then incubated at 37 °C for 24–48 h, colonies selected and then characterized. Nutrient broth medium was prepared by dissolving 2.8 g of nutrient broth powder in 400 mL of distilled water and sterilized by autoclaving at 121 °C for 15 min. One bacterial colony was picked from the plate by sterile inoculating loop and inoculated aseptically into sterile nutrient broth and incubated at 37 °C for 24 h to obtain actively growing culture. Gram stain was performed to determine the morphological and Gram reaction characteristics of the bacterium. A drop of bacterial broth culture was placed on the clean glass slide, allowed to air dry, and heat-fixed by passing the slide through the top of the flame. The slide was then subjected to 1 min of crystal violet stain, followed by rinsing with distilled water. The smear was then treated with the gram iodine solution for 1 min to form the crystal violet-iodine complex. The smear was decolorized using 95% ethanol until the ethanol ran clear and was then rinsed with distilled water. The smear was then counterstained with safranin for 30–60 s before being rinsed and air-dried. The slides were then viewed under a light microscope with an oil-immersion objective lens (100x) to visualize the bacteria and determine the Gram reaction, with the Gram-positive bacteria appearing purple due to retention of the crystal violet, and the Gram-negative bacteria appearing pink or red due to the safranin. The results showed that the water bodies sampled exhibited the presence of both Gram-positive and Gram-negative bacteria, which indicated the phenotypically heterogeneous cultivable bacterial populations in the rural water system. Nevertheless, the culture-based and Gram-staining method used show some limitations in that they do not offer species identification nor provide complete characterization of the microbial community.

### 2.4 DNA extraction

CTAB (cetyltrimethylammonium bromide) extraction is a common means for isolating genomic DNA from environmental samples. Total microbial DNA was extracted using the CTAB extraction method. The CTAB lysis buffer was prepared by mixing 2% CTAB, 100 mM TrisHCl, 20 mM EDTA and 1.4 M NaCl, and 20 mg mL^–1^ Proteinase K was added to the buffer to lyse the cells and digest proteins. A total of 600 μL of CTAB lysis buffer and 20 μL of Proteinase K were mixed well and incubated for 45 min at 65 °C in an iTHERM D150-1 dry bath (Neuation, India) to break down the microbial cell wall and release intracellular DNA from the cells. After incubation, the lysate was treated with equal volume (v/v) chloroform-isoamyl alcohol (24:1) to extract the proteins and debris, followed by centrifugation in the iFUGE MT15R centrifuge (Neuation, India), at 12,000 rpm for 10 min to separate the water and organic phases. The aqueous phase containing the isolated DNA was transferred into a new, sterile microcentrifuge tube and the DNA was precipitated out of the aqueous phase by the addition of 0.6 volumes of cold isopropanol. Incubation was continued for 20 min at -20 °C, followed by centrifugation at 12,000 rpm for 10 min in the same centrifuge, and was used to obtain the DNA pellet that was now visible to the naked eye. The purified DNA pellet was then washed with 70% ethanol to remove salt and contaminants, air-dried under sterile conditions, then finally dissolved in 30–50 μL of nuclease-free water and stored at -20 °C until needed for molecular analysis. This isolated DNA was then PCR increased to obtain class 1 integrase (*intI1*) gene fragments. This DNA was taken from the water matrix rather than the microorganisms on microplastic surfaces.

### 2.5 PCR amplification of the *intI1* antibiotic-resistance-associated marker

The polymerase chain reaction (PCR) was performed to detect the *intI1* antibiotic-resistance-associated marker associated with class 1 integrons, which is often used as an environmental marker of anthropogenic influence and mobile genetic elements such as integrons. PCR was not used in a quantitative manner to determine the concentration of the class 1 integrase gene, but rather to qualitatively screen for its presence within the sample. A 280 bp fragment of the *intI1* gene was increased by PCR using the *intI1*-F forward (CCTCCCGCACGATGATC) and *intI1*-R reverse (TCCACGCATCGTCAGGC) primers as described by Goldstein et al. (2001). Detection of the *intI1* marker by conventional PCR is used as a qualitative indicator (not quantitative) of its presence in the environmental sample DNA, but this result is not used as an indicator of phenotypically antibiotic resistant bacteria, specific antibiotic resistance genes, or antibiotic resistance levels. A 20 μL *intI1* PCR reaction was prepared by mixing 10 μL of 2 × Red Dye Master Mix, 1 μL of forward primer, 1 μL of reverse primer, 1 μL of extracted template DNA, and 7 μL of nuclease-free water. All PCR setups were performed in a P-Lab PCR hood (Precision Scientific) and PCR tubes were prepared using appropriate Finnpipette F2 micropipettes (Thermo Scientific) and mixed in an iSWIX JR VT NXT vortex mixer (Neuation, India). Amplification was performed in a 96-well thermal cycler (GeNei, India). The PCR protocol included an initial denaturation step at 95 °C for 5 min, followed by 35 cycles of denaturation at 95 °C for 30 sec, annealing at 55 °C for 30 s and extension at 72 °C for 30 s and a final extension step at 72 °C for 5 min to fully increase the target DNA fragments. A no-template control (NTC) containing all the PCR components without template DNA (replaced with nuclease-free water) was increased under the same conditions to rule out possible contamination by reagents or procedures. All the increased PCR products contained the expected *intI1* gene fragment, as determined by agarose gel electrophoresis analysis of the PCR products.

### 2.6 Agarose gel electrophoresis

Confirmation of the amplification of was done by agarose gel electrophoresis. Preparation of the agarose gel for the electrophoresis process involved mixing the required concentration of agarose with the electrophoresis buffer, pouring the molten gel mixture into the gel tray, and allowing it to strengthen. PCR products were loaded into the gel wells along with a 50 bp DNA ladder used as a molecular size marker. Electrophoresis was conducted under standard conditions using an Orange Power Supply Basic Dual to separate DNA fragments by molecular size. After electrophoresis, the gels were visualized under ultraviolet illumination using Orange UV transilluminator (India). The presence of the distinct band at approx. 280 bp indicated that the *intI1* marker fragment had been successfully increased and was interpreted as qualitative evidence of the presence of the marker in the analyzed environmental DNA samples only.

### 2.7 Statistical and spatial analysis

Means, standard deviations, medians, and minimum and maximum values were calculated for turbidity and relative polymer composition. Data normality was tested with the Shapiro-Wilk test. The homogeneity of variances was estimated with Levene’s test. Because of non-normality and differences in sample sizes across water categories, differences between drinking water, tap water, wastewater and river water for the respective variable were assessed with the non-parametric Kruskal-Wallis test (*p* < 0.05). Spearman’s rank correlation coefficient (ρ) was calculated to evaluate the association between turbidity to relative polymer composition. The fact that the sum of the PE+PP and PP percentages of a variable was equal to 100% meant that the inverse relationship between those two was interpreted merely as a mathematical dependence, and not as an association in the environmental context.

Hierarchical Cluster Analysis (HCA) was used to visualize similarities between sample sites with an exploratory multivariate approach using standardized variables, Ward’s method of linkage, and Euclidean distance measure. This classification should be interpreted with caution because of small and unbalanced sample sizes, particularly for the two river water samples. Spatial statistical analysis was performed using ArcGIS. The relative polymer composition values were analyzed for spatial autocorrelation using Global Moran’s I and spatial hotspots and coldspots were identified with Getis-Ord Gi* statistic. Exploratory analysis of spatial distribution was performed using the IDW interpolation method. After IDW interpolation, the output of the precipitation maps was evaluated through leave-one-out cross-validation in ArcGIS Geostatistical Analyst. For this purpose, the mean prediction error was calculated to assess bias and the root mean square error (RMSE) to assess uncertainty. Due to sample size limitations, the interpolated surfaces are interpreted as exploratory spatial patterns, not predicted expected values at unsampled locations. Spatial autocorrelation and hotspot analyzes were not performed on the river water category due to the low number of sampling locations (*n* = 2).

## 3 Results and discussion

### 3.1 FTIR characterization of microplastics in drinking water

The FTIR spectra of the microplastics isolated from drinking water samples are shown in Figures 3a–e. This suggests the presence of characteristic absorbance peaks of the polyolefins, specifically polyethylene (PE) and polypropylene (PP). Besides the absorption bands mentioned above, in the region between 2,850 and 2,950 cm^–1^ peaks corresponding to the stretching vibrations of C-H bonds of aliphatic hydrocarbons have been observed; these peaks clearly indicate the presence of polyolefin polymer backbones (Sangkham et al., 2026). Clear bands in the range 1,380–1,460 cm^–1^ indicate the presence of CH_3_ bending vibrations of polypropylene (Volova et al., 2025).

**FIGURE 3:**
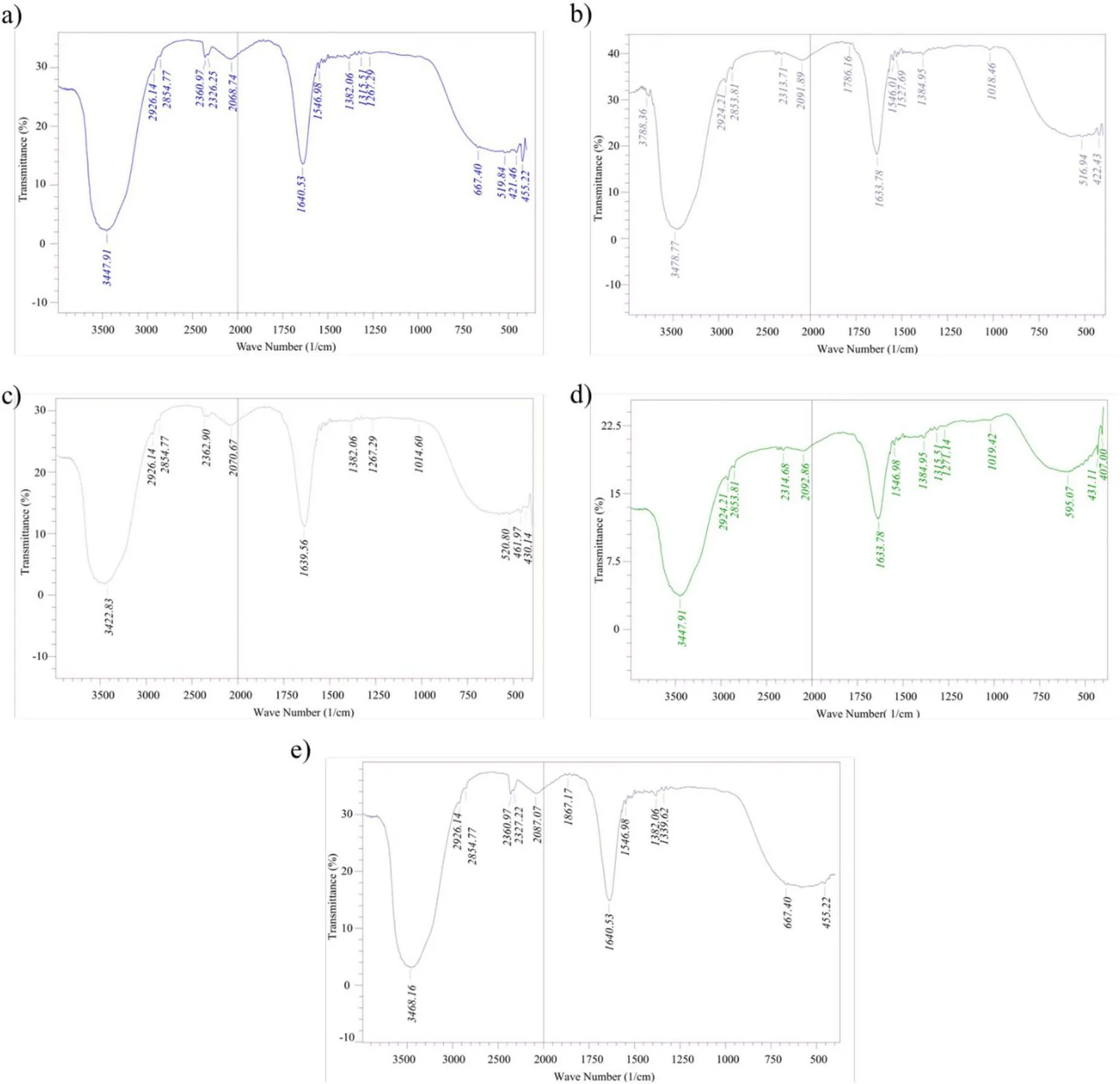
FTIR spectra of separated MP particles in drinking water from five sampling sites. **(a)** DS-1, **(b)** DS-2, **(c)** DS-3, **(d)** DS-4, and **(e)** DS-5. Characteristic absorption bands of polyolefin polymers are shown in all spectra.

The broad absorption bands around 3,400 cm^–1^ from O-H stretching vibrations confirm the presence of moisture or surface oxidation of the microplastic particles (Abu Shuheil et al., 2026; Dai et al., 2023). Further analysis of the spectra found differences between the two sites in the spectral observations at certain wavelengths. The strong peaks at 2,926 and 1,382 cm^–1^ shown in Figures 3a,e confirmed PP as the major polyolefin polymer (Blanco et al., 2025).

The existence of mixed PE and PP was affirmed by the strong CH_3_ bending and C-H stretching vibrations seen in the FTIR spectra as shown in Figure 3b. However, the peaks were less intense in Figure 3c, indicating a lower spectral response of the detected polyolefin peaks. Absorption bands corresponding to polymeric signatures were narrower and more defined in the sample shown in Figure 3d. Polyolefin-related absorption bands could be identified in the spectrum of all samples. Thus, the results indicate that most of the identified microplastic particles were PE and PP related signatures typically associated with packaging, pipelines and plastic containers. The spectral similarity and variation in peak height suggest that polyolefin microplastics were present in the drinking water samples, but varied by sampling site which is likely due to the source water quality and the circumstances where sampling took place.

### 3.2 FTIR characterization of microplastics in tap water

The FTIR spectra of the microplastic particles that were isolated from the tap water samples TS-1, TS-2, TS-3, and TS-5 show characteristic absorption peaks belonging to polyolefin-based polymers, mainly PE and PP. Aliphatic C-H stretching vibrations from 2,850 to 2,950 cm^–1^ present in the FTIR spectra of all samples represent the prevalence of hydrocarbon polymer backbones in the microplastic compositions as presented in Figures 4a–d. The CH3 bending vibrations in the wavenumber region of 1,380–1,460 cm^–1^ indicate the existence of polypropylene structures in the FTIR spectra. Broad absorption bands were observed in the region around 3,400 cm^–1^ due to OH stretching vibrations from moisture adsorption or surface oxidation. Spectra of the various tap water samples were different. The C-H stretching and CH3 bending peaks were very strong in sample TS-1 as shown in Figure 4a, which contained a mixture of PE and PP. The absorption bands of the PP section in the sample TS-2 as given in Figure 4b are relatively stronger, indicating a higher contribution of PP in this sample. In the sample TS-3 are presented in Figure 4c, the peaks are less intense, in other words, polyolefinic signatures are less prominent. Conversely, for sample TS-5 as given in Figure 4d, the well-defined peaks indicate a sharper definition of the identified polymer signatures present. The spectrum of TS-4 (which had the same water supply source as DS-4) was almost identical to that of DS-4 confirming that the same polymers that were present in the water at the point of supply were also present in the distributed water at the sampling point. Nevertheless, variability in other tap water samples suggests that site level differences in polymer compositions may also be due to the source water composition, or differences in distribution systems, storage conditions, or handling conditions; the scope of the study does not explore these differences.

**FIGURE 4:**
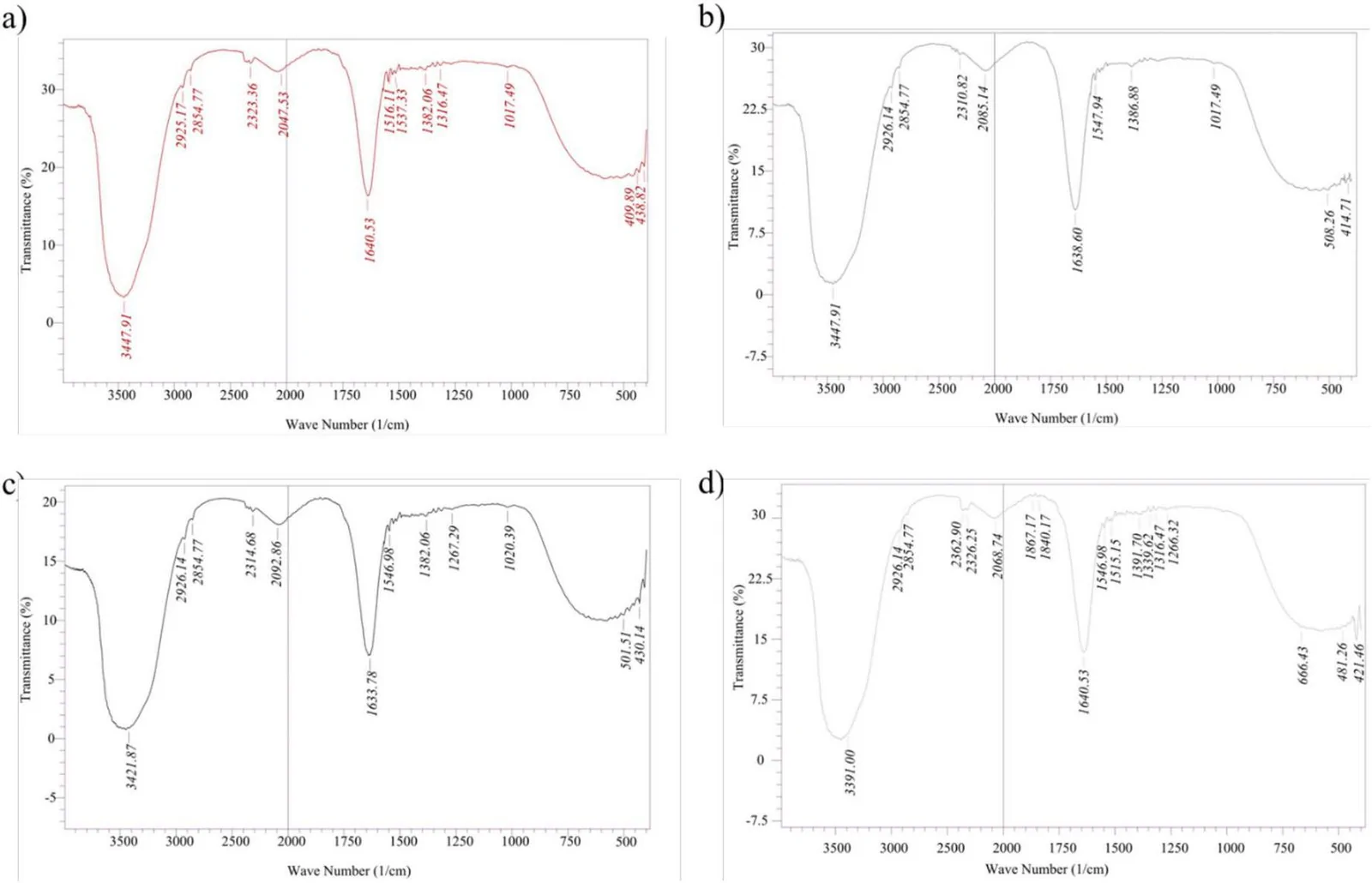
FTIR spectra of microplastic particles isolated from tap water samples collected at 4 sampling sites **(a)** TS-1, **(b)** TS-2, **(c)** TS-3 and **(d)** TS-5) and their typical absorption bands for PP and PE polymers, are shown under each panel.

### 3.3 FTIR characterization of microplastics in wastewater

FTIR spectra of MP particles shown in Figures 5a–e recovered from wastewater samples exhibit absorption peaks of C-H stretching vibrations between 2,850 and 2,950 cm^–1^, suggesting the presence of hydrocarbon polymer chains in the MP particles. Unique absorption peaks between 1,380 and 1,460 cm^–1^ in wastewater samples indicate microplastics of PP as it shows CH_3_ bending vibrations. Absorption peaks between 1,600 and 1,650 cm^–1^ and broad absorption peaks between ∼3,400 cm^–1^ exhibited greater intensity in the spectra of waste water samples than drinking and tap water samples indicating increased oxidization, humidity, and external attachment of organic molecules on the polymeric surface of the microplastics. In addition, there are differences between the spectra, indicating the heterogeneity of the sample and increasing degree of polymerization with increasing complexity of the wastewater (Figure 5). As shown in Figures 5a,e, the polyolefin absorption bands are well defined and highly pronounced; however, the peaks in Figure 5b are broader and somewhat distorted, which may be due to environmental aging or composite polymer structures. Moderate intensity peaks of polyolefin characteristic bands were observed in Figure 5c whereas sharper peaks and other minor absorption bands in Figure 5d indicated possible interaction with the organic/inorganic contaminants present in the wastewater

**FIGURE 5:**
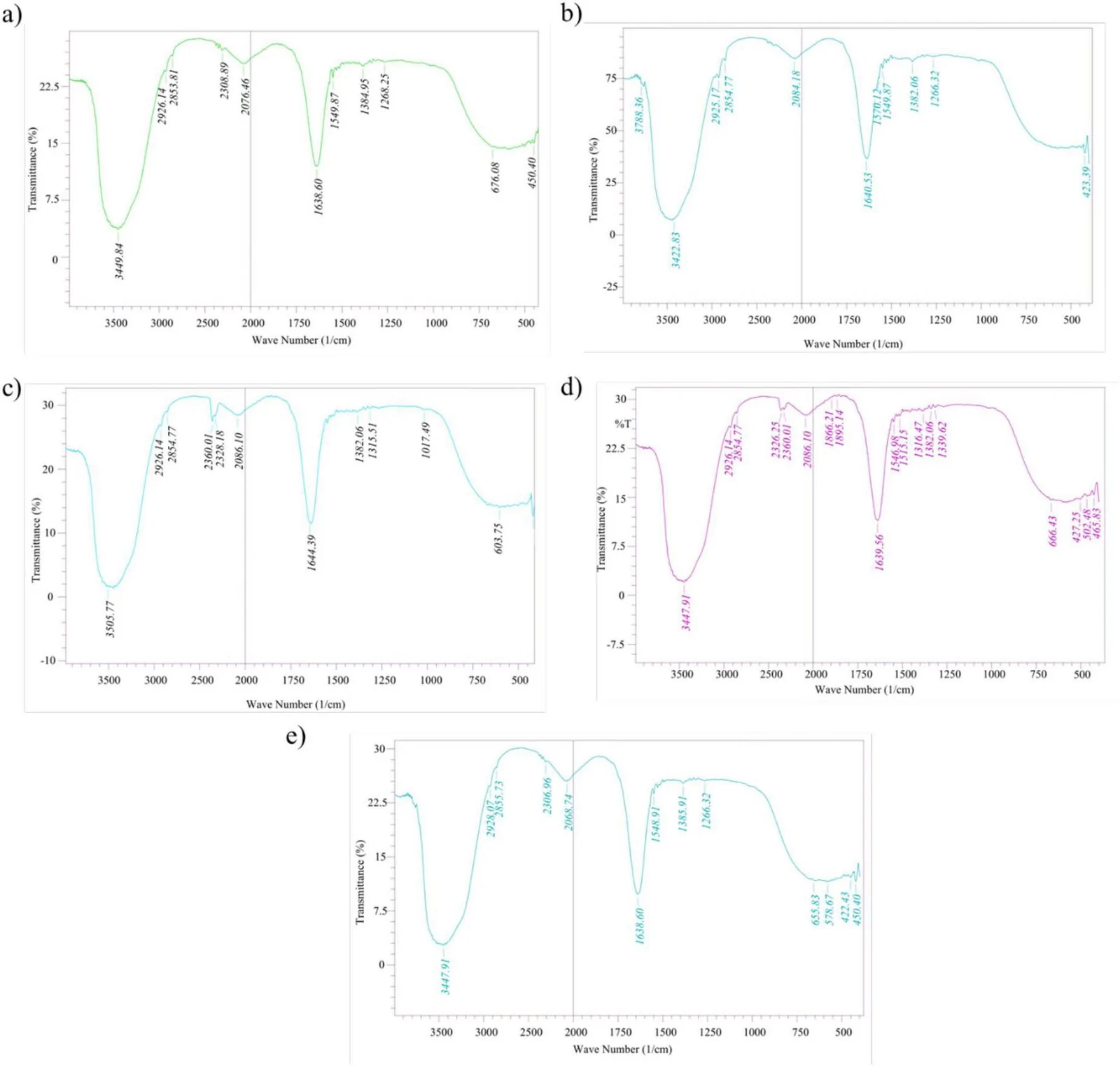
FTIR spectra of microplastic particles isolated from wastewater samples collected at five locations **(a)** WS-1, **(b)** WS-2, **(c)** WS-3, **(d)** WS-4 and **(e)** WS-5, showing characteristic selective absorption bands of polyolefin polymer constituents.

Wastewater spectra displayed higher peak intensity, broader absorption bands, and larger spectral variation than spectra from drinking and tap water. Differences in the spectral characteristics of wastewater particles are likely attributed to heterogeneous anthropogenic inputs and the complex organic matrices of wastewater. The consistently observed polyolefin-associated absorption bands in all wastewater samples confirmed the presence of PE- and PP-associated spectra amongst the wastewater particles. The addition of spectral complexity indicates that microplastic particles are being subjected to processes (fragmentation and surface modification) in the wastewater treatment plants that may cause changes along their life cycle. As such, wastewater systems may be important sinks for microplastics, and these transformations may influence microbial colonization, polymer adsorption of contaminants, and the transport and persistence of microplastic-associated contaminants in the environment.

### 3.4 FTIR characterization of microplastics in river water

Figures 6a,b presents the FTIR spectra of microplastic particles isolated from river water samples. The spectra exhibited characteristic absorption bands associated with polyolefin-based polymers. Prominent absorption peaks observed in the range of 2,850–2,950 cm^–1^ correspond to aliphatic C-H stretching vibrations, confirming the presence of hydrocarbon polymer backbones. In addition, absorption bands observed in the range of 1,380–1,460 cm^–1^ are attributed to CH_3_ bending vibrations, indicating the presence of PP. There are also broad absorption bands around 3,400 cm^–1^ and moderate peaks around the area of 1,600–1,650 cm^–1^, which indicate the interaction of moisture and partial oxidation of the microplastic particles on its surface.

**FIGURE 6:**
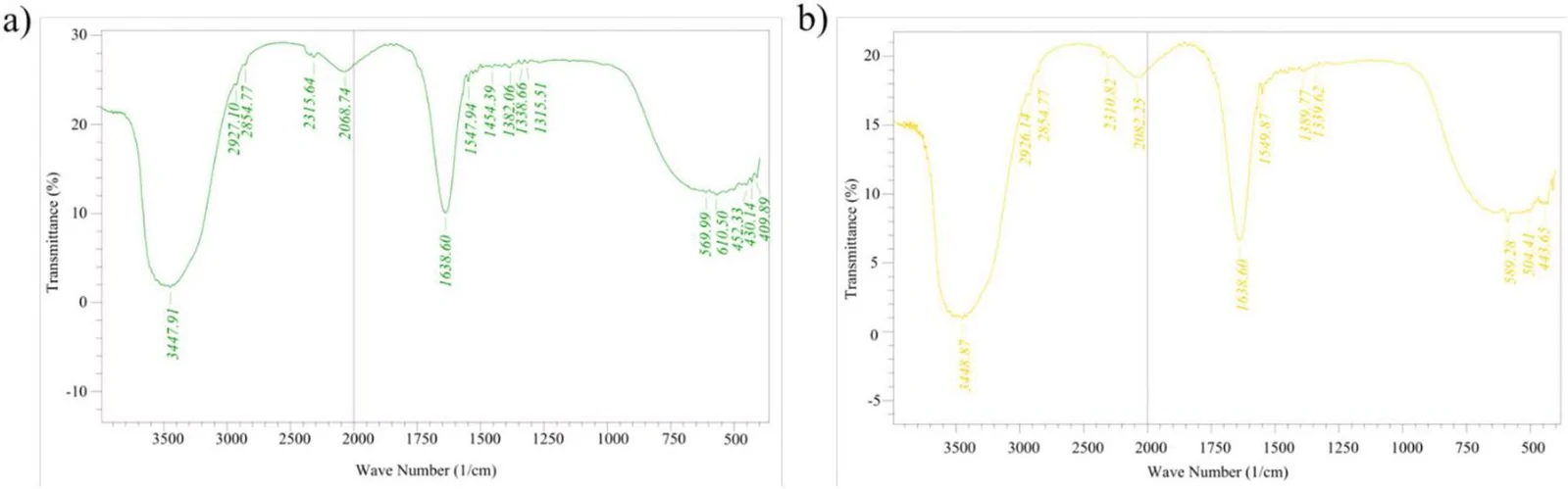
FTIR spectra of microplastic particles isolated from river water samples collected at two sampling locations: **(a)** RS-1 and **(b)** RS-2, showing characteristic absorption bands associated with polyolefin polymers.

Comparative analysis of the two spectra indicates variations in polymer characteristics and peak intensity between the sampling locations. Figure 6a exhibits relatively stronger and more distinct polyolefin absorption bands, suggesting a stronger spectral response or a comparatively less altered polymer structure. On the other hand, the peaks in the river water spectra as given in Figure 6b are wider, less pronounced, and could also be due to weathering of the polymers in the water, dilution, physical aging of the material, and mixing of different types of polymers in transport. In addition, compared to the spectra from wastewater samples, the river peaks are lower and the curve is smoother, which can be attributed to environmental weathering and variations in particle composition. Despite this, the presence of PE and PP absorption bands in the river water samples implies that the polyolefin signals arising from the PE- and PP-mediated polyolefins were present. Due to only two river water samples being collected, these spectral differences are interpreted as site-specific differences rather than regionally coherent river system trends.

### 3.5 Polymer composition and spatial distribution of microplastics in drinking water

The qualitative findings for microplastics and their relative polymer composition found in the collected drinking water samples are summarized in Figure 7 and Table 1. Microplastics were found in all collected water sampling sites, and plastic debris and turbidity (0 NTU), as indirect indicators for microplastics contamination, were not observed in collected water samples. These results further illustrate how conventional evaluations of the data can miss the true relationships and point to the merit of advanced analytical techniques, such as FTIR spectroscopy. While the variations in polymer composition for the sampling points were heterogeneous, PP was the most relevant polymer fraction in different sampling points. Sample 5 showed a 100% proportion of PP, whereas the proportion of PP fraction was large in Samples 2 and 3, 74.71 and 88%, respectively, and the proportion of mixed PE + PP fractions was predominant in Sample 4, 93.86%. These results suggest that the polymer composition is different at the two sites. For Sample 1, the polymer composition was almost the same, namely 66.67% PE+PP and 33.33% PP. The polymer imaging results are similar to the FTIR spectral analysis. As we mentioned in section 3.1, the presence of the polyolefin-based polymers was identified in all the samples by the characteristic bands of C-H stretching in the range 2,850–2,950 cm^–1^ and CH_3_ bending vibrations in the range 1,380–1,460 cm^–1^ in FTIR analysis.

**FIGURE 7:**
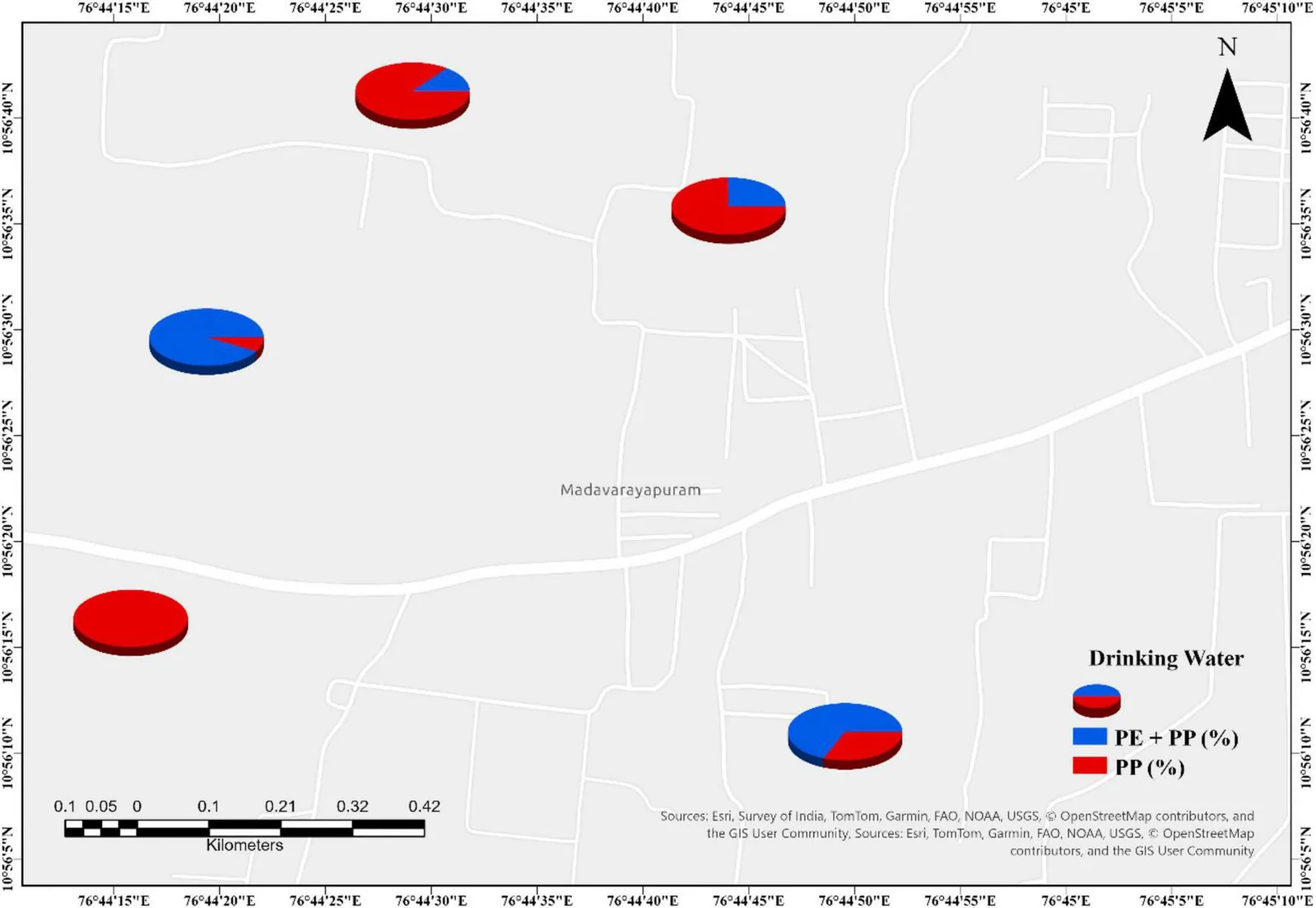
Relative composition and spatial distribution of microplastic polymers in drinking water samples across the study area, illustrating variations in PE and PP fractions among sampling locations.

**TABLE 1:** Geographic coordinates, field observations, and relative polymer composition of microplastics identified in drinking water samples across the study area.

Sample ID	Lat	Long	Visible plastic present	Turbidity	PE+PP (%)	PP (%)
**DS-1**	10.93639	76.7471	No	0	66.67	33.33
**DS-2**	10.94329	76.74557	No	0	25.29	74.71
**DS-3**	10.9448	76.74142	No	0	12	88
**DS-4**	10.94157	76.73872	No	0	93.86	6.14
**DS-5**	10.93788	76.73772	No	0	0	100

Spatial mapping also demonstrated that PP-dominant relative polymer compositions were recorded at more sites than samples with mixed PE+PP composition, suggesting the influence of local contamination sources, such as household plastics, containers used for storage, and distribution networks. The increased prevalence of PP is consistent with its common application in household plastic products, such as packaging and water storage containers. This close correspondence between the polymer composition of spatial mapping results and FTIR-based identification suggests that the differences in polymer composition observed on fields may be due to a combination of heterogeneous plastic sources. That polymer was detected in the absence of turbidity or other visible particulate plastic at all tested sampling locations suggests that microplastics could occur in the absence of customary visual or turbidity indicators. The high level of variability over short distances may indicate effects of local environments and infrastructure as well as those of more distant sources. These site-specific differences should therefore be seen only as descriptive spatial heterogeneity and not as evidence of statistically specific contamination sources, as would be the case if those sources were identified in the present work. The spatial and chemical results combined show that drinking water supplies may be susceptible to microplastic contamination under low levels of turbidity, suggesting that monitoring programs need to be broader.

### 3.6 Polymer composition and spatial distribution of microplastics in tap water

The spatial distribution of microplastics in tap water and the percentage of total microplastics within each size category are seen in Figure 8 and Table 2. Microplastics were found in all sites, including sites with low turbidity levels, suggesting that the presence of microplastics in the tap water distribution system is not reflective of turbidity levels. Overall, more spatial variability in polymer relative composition across sampling locations was found in tap water samples than in drinking water samples. PE+PP was the most dominant polymer class at most sampling locations, although PP was improved at some locations. Sample 3 was 100% PP, suggesting local PP-dominance, while Samples 2 and 4 had prominent PE (95.73%) and PP (93.86%) respectively, suggesting mixed polymer inputs. Sample 1, with moderate turbidity (4.5 NTU), had a composition of 76.47% PE+PP and 23.53% PP. Sample 5, with similar turbidity, had a relatively balanced composition of 56.52% PE+PP and 43.48% PP. The spatial trends in composition correspond to findings on polymer composition using FTIR in section 3.2.

**FIGURE 8:**
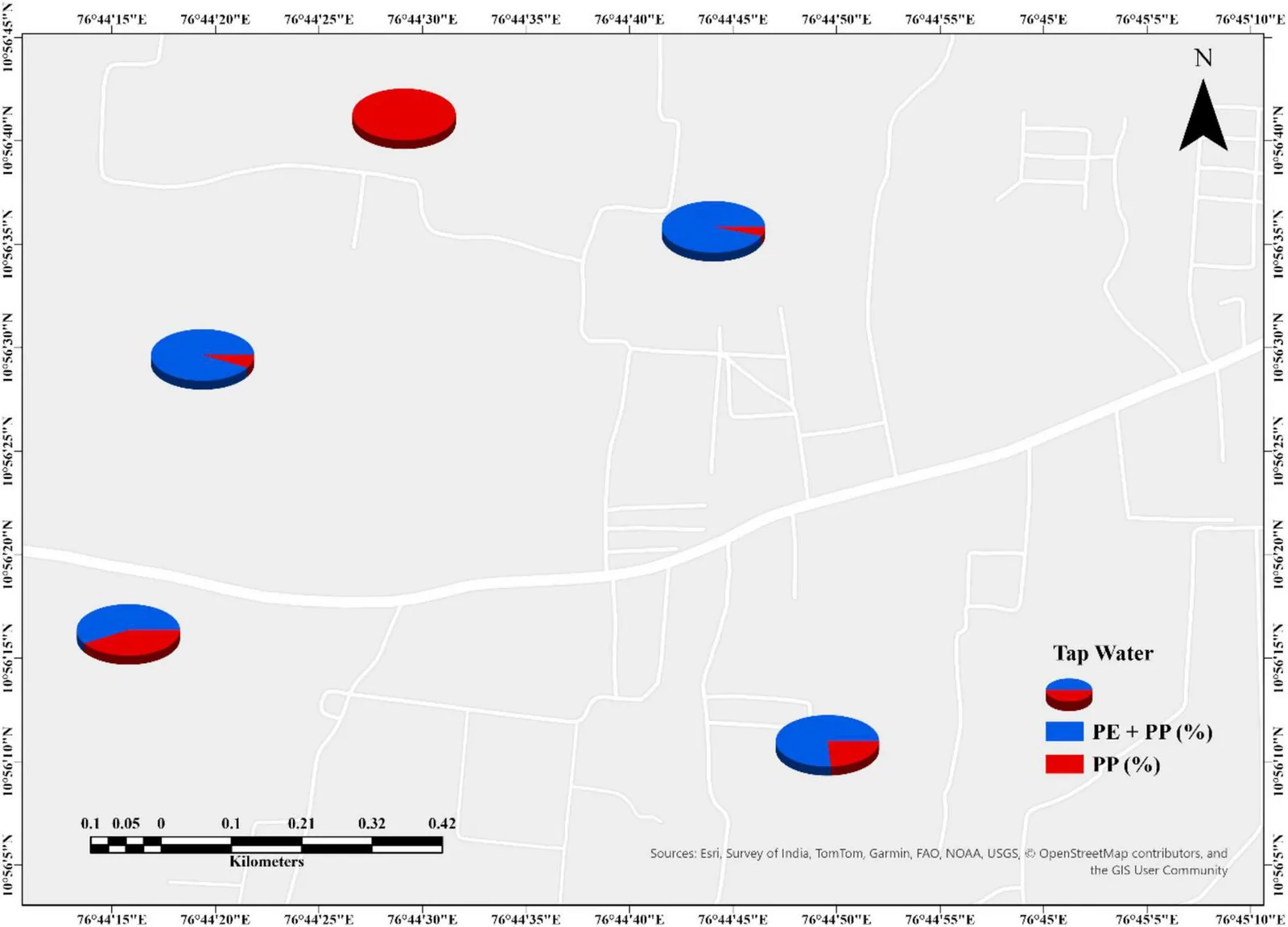
Spatial distribution and relative composition of microplastic polymers obtained from tap water samples in the study area; PE and PP fractions differed among the sampling locations.

**TABLE 2:** Geographic coordinates, field observations, and relative polymer composition of microplastics identified in tap water samples across the study area.

Sample ID	Lat	Long	Visible plastic present	Turbidity	PE + PP (%)	PP (%)
TP-1	10.93639	76.7471	No	4.5	76.47	23.53
TP-2	10.94329	76.74557	No	0	95.73	4.27
TP-3	10.9448	76.74142	No	0	0	100
TP-4	10.94157	76.73872	No	0	93.86	6.14
TP-5	10.93788	76.73772	No	0	56.52	43.48

Sample TS-4 had the same spectral signals as DS-4 since both samples came from the same source water, showing that the same polymer was being removed from the source water and tap water during sampling, and that the sampling and analytical methods are consistent and reproducible. Differences in other tap water samples may be due to differences in source water quality, distribution system, or local conditions in the leaching/abrasion of plastic distribution and consumer storage infrastructure (e.g., plastic pipes, storage tanks, fittings, containers) or with stagnation effects. One possible influence on these distributions that was not tested in this study is the infrastructure and usage at geographically nearby sampling sites. Particularly, the high proportion of PP at some sites may be caused by polypropylene plumbing or storage systems, or the presence of PE-dominated or mixed polymer profiles may have been due to the presence of multiple sources at these sites. The detection of microplastics in all samples, regardless of their turbidity level, further confirms that their presence was not consistently due to the turbidity in the tap water samples.

### 3.7 Polymer composition and spatial distribution of microplastics in wastewater

The relative polymer composition rates and spatial distributions of the examined MP particles among all collected wastewaters can be seen in Figure 9, and the MP polymer compositions in Table 3. All of the wastewater sampling sites collected plastic debris, and turbidity values (0–14.3 NTU) were much higher when compared to drinking water and tap water, indicating anthropogenic pollutants. Plastic particles were present in all samples indicating that wastewater environments may be important areas for the accumulation/input of plastic derived microplastics. With respect to spatial heterogeneity in polymer composition, mixed polyolefin (PE + PP) and PP-dominant profiles were common across sites; however, considerable differences in polymer profiles were identified across some sampling sites. The composition of samples 1 and 2 was similar (61.11% polyolefin fraction + 38.89% PP fraction and 54.22% polyolefin fraction + 45.78% PP fraction, respectively). However, sample 3 is associated with a higher polyolefin fraction (86.79%) and a lower PP fraction. Thus, the local composition was PE+PP-dominant. Sample 4 had high polyolefin fraction (81.58%) and no turbidity, indicating that microplastics are not always associated with turbidity. Sample 5 was composed entirely of PP, which suggests that PP was locally dominant in the water column. The spatial patterns are in agreement with the results from the FTIR spectral analysis in section 3.3, which indicates that polyolefin polymers are the predominant polymer in the wastewater.

**FIGURE 9:**
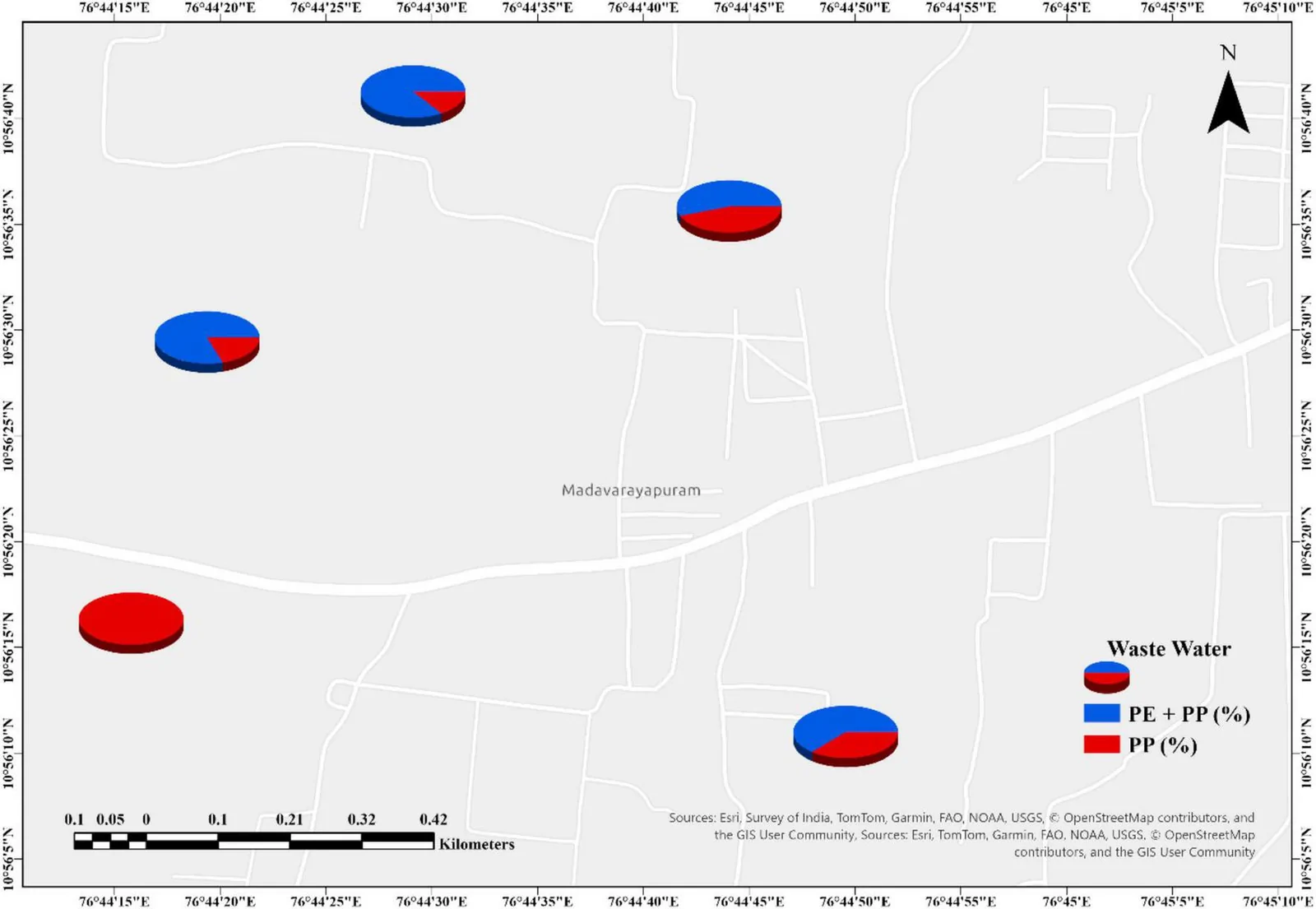
Spatial distribution and relative polymer composition of microplastic polymers in different wastewater sampling sites across the study area, showing variations in the relative fractions of PE and PP.

**TABLE 3:** Geographic coordinates, turbidity, visible plastic observations, and relative polymer composition of microplastics identified in wastewater samples collected from sites in the study area.

Sample ID	Lat	Long	Visible plastic present	Turbidity	PE + PP (%)	PP (%)
**WS-1**	10.93639	76.7471	Yes	4.7	61.11	38.89
**WS-2**	10.94329	76.74557	Yes	10.9	54.22	45.78
**WS-3**	10.9448	76.74142	Yes	14.3	86.79	13.21
**WS-4**	10.94157	76.73872	Yes	0	81.58	18.42
**WS-5**	10.93788	76.73772	Yes	4.1	0	100

Spatial heterogeneity of relative polymer composition among wastewater sample may indicate anthropogenic pressure, e.g., household waste disposal, plastic litter, graywater discharge, surface runoffs from developed environments. Polyolefin-rich wastewater samples may indicate plastic films, carry bags, and fragmented and compacted plastic packaging. Higher relative PP fractions at some wastewater sites may indicate higher amounts of PP-dominant polymer compositions of polymers entering those sites, but existing data cannot directly attribute plastic sources to individual sites. This suggests that areas where wastewater contains increased visible plastic fragments, turbidity, and varying polymer compositions would be potential sites for microplastic accumulation and breakdown within the study area. The presence of microplastics in Sample 4 at a low turbidity would indicate that the contamination of our wastewater samples is not simply a function of suspended particulate matter, but is influenced by localized inputs, fragmentation and retention of microplastics throughout the wastewater treatment process. While distributions and polymer typing indicate site-specific heterogeneity in observed microplastic concentrations at wastewater sites, these patterns do not show statistically important microplastic pollution hotspots.

Wastewater systems are more internally heterogeneous than drinking and tap water systems, with direct anthropogenic loading of plastics from household waste, plastic dumping, graywater and wash-off from urban surfaces. A high fraction of PE+PP is indicative of plastic films, carry bags, and aged and degraded packaging products, whilst a high fraction of PP is suggested to be associated with single-use rigid plastic items, containers and packaging materials, and household waste. Based on the presence of visible plastic debris and turbidity in most samples, wastewater environments may act as local sinks and transport pathways for microplastics; however, the contributions of local sources were not quantified in this study.

### 3.8 Polymer composition and spatial distribution of microplastics in river water

Microplastics were found in both water samples as given in Figure 10 and Table 4 for characteristics and relative polymer composition of microplastics. Plastic material was also found on both sampling sites. Microplastics were detected at both sampling locations, with visible plastic debris observed at each site, indicating the presence of plastic contamination within the investigated river locations. River water samples exhibited lower turbidity (0–1.66 NTU) compared with wastewater systems, although the present dataset does not permit direct attribution of this difference to dilution or hydrodynamic processes. Polymer composition varied considerably between the two sampling locations. Sample 6 showed strong dominance of PP, with a fraction of 80.65% and a mixed polyolefin (PE + PP) fraction of 19.35%, indicating a locally PP-dominant composition. In contrast, Sample 7 exhibited a markedly different composition, with a polyolefin fraction of 94.29% and a comparatively low PP fraction of 5.71%, indicating a locally PE+PP-dominant composition. The pronounced variation between two geographically proximate sampling sites indicates considerable site-specific heterogeneity; however, interpretation should be made cautiously because only two river water samples were available. These spatial patterns are consistent with the FTIR spectral analysis presented in section 3.4. Compared with the wastewater samples, the FTIR spectra of the river water are lower in peak intensity and less distinct in spectral signal, indicating differing particle characteristics and environmental exposures.

**FIGURE 10:**
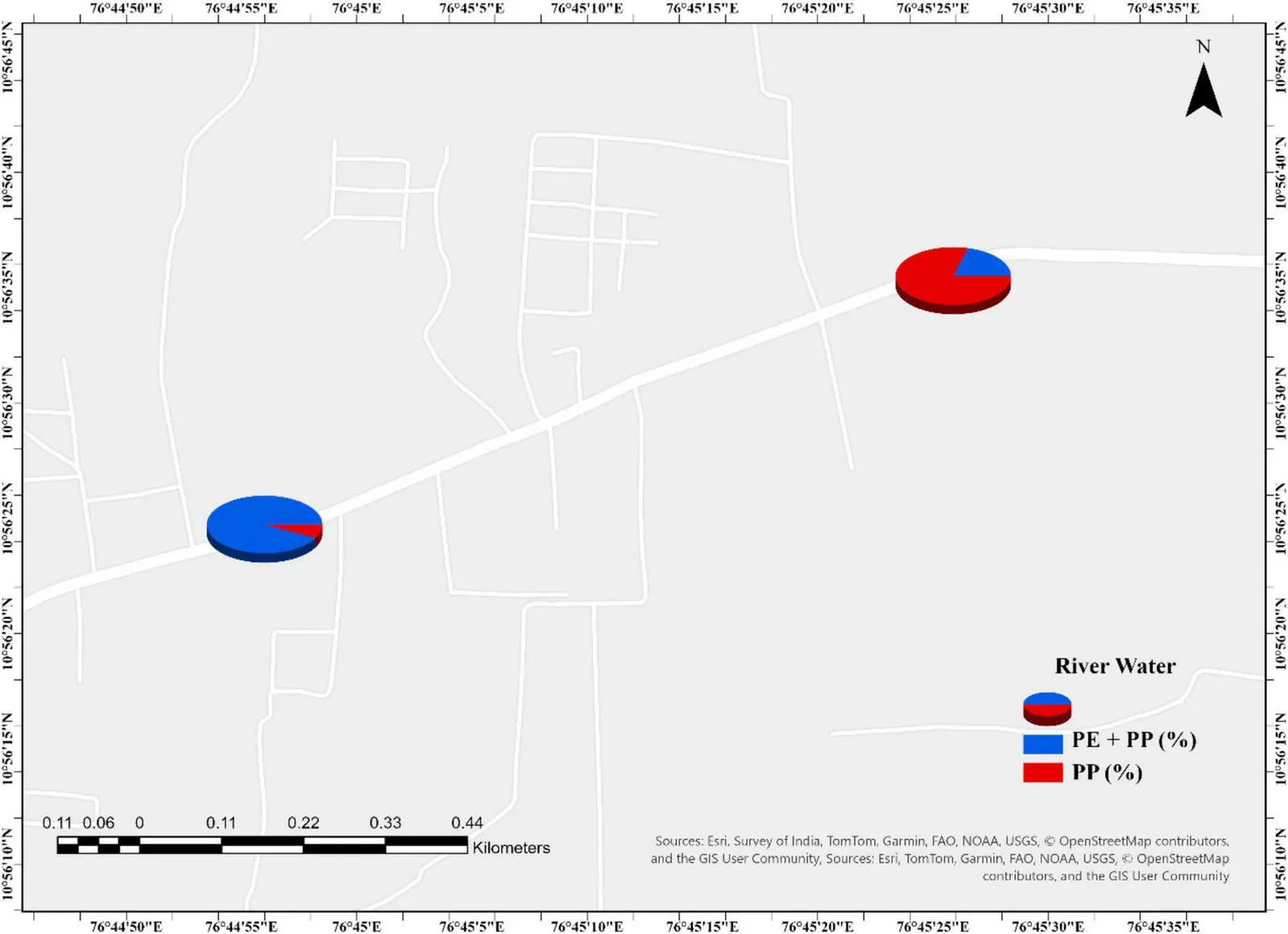
Spatial distribution and relative composition of microplastic polymers sampled in river water across the study area. There is variability among sampling locations in the contributions of PE and PP.

**TABLE 4:** Geographic coordinates, turbidity values, visible plastic observations, and relative polymer composition in microplastics in the river water samples at each site.

Sample ID	Lat	Long	Visible plastic present	Turbidity	PE + PP (%)	PP (%)
**RS-1**	10.9432	76.75719	Yes	1.66	19.35	80.65
**RS-2**	10.9402	76.74889	Yes	0	94.29	5.71

The lack of spatial trends and the presence of PP-dominant and mixed polyolefin-rich samples indicate that polymer composition at river water was site specific, resulting in limited spatial trends in polyolefin deposition. Microplastics were found in low-turbidity conditions, suggesting that particulate pollution was not the only mechanism of microplastics moving from the source. However, this study does not prove direct microplastic transport from source to river via this pathway; river systems could be a transport location. Despite the spatial mapping results closely paralleling FTIR polymer identification patterns, the limited number of river water samples collected provided little chance of generalizing findings across space or environmental conditions.

### 3.9 Spatial interpolation and comparative analysis of microplastic distribution

Figures 11, 12 are interpolated maps that show the relative polymer composition of the PE+PP polymer and the PP polymer, respectively. The maps were plotted using the IDW interpolation method and compare the relative polymer compositions of drinking water, tap water, wastewater, and river water systems. In this way, interpolated outputs provided continuous, spatial predictions of relative polymer composition, which could be used to visualize exploratory spatial distributions between sampling sites, but not to predict absolute microplastic concentrations or particle abundances. Figure 11 shows the relative abundances of PE+PP fractions sampled at each of the study water bodies. For wastewater systems, the relative fraction of PE+PP was variable both spatially and within tap water systems. For drinking water systems, substantial spatial variability in both high and low relative fraction of PE+PP was seen. The polymer composition of river water systems at these two locations was also different but, given the small number of samples taken at each of these two locations, these values should be treated with caution.

**FIGURE 11:**
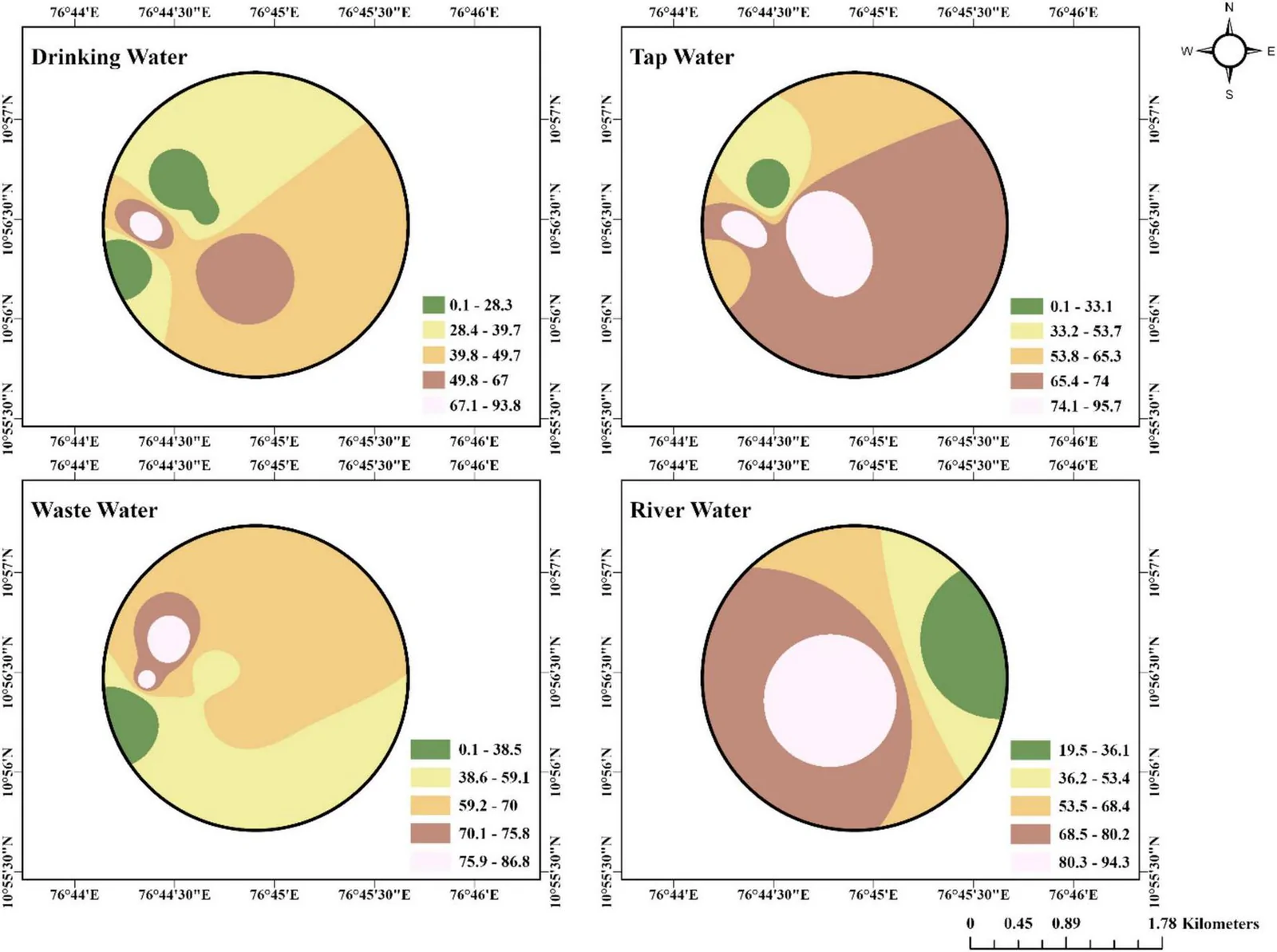
Comparative interpolated spatial distribution of relative mixed polyethylene and polypropylene (PE+PP) composition across drinking water, tap water, wastewater, and river water systems, generated using the IDW interpolation method.

**FIGURE 12:**
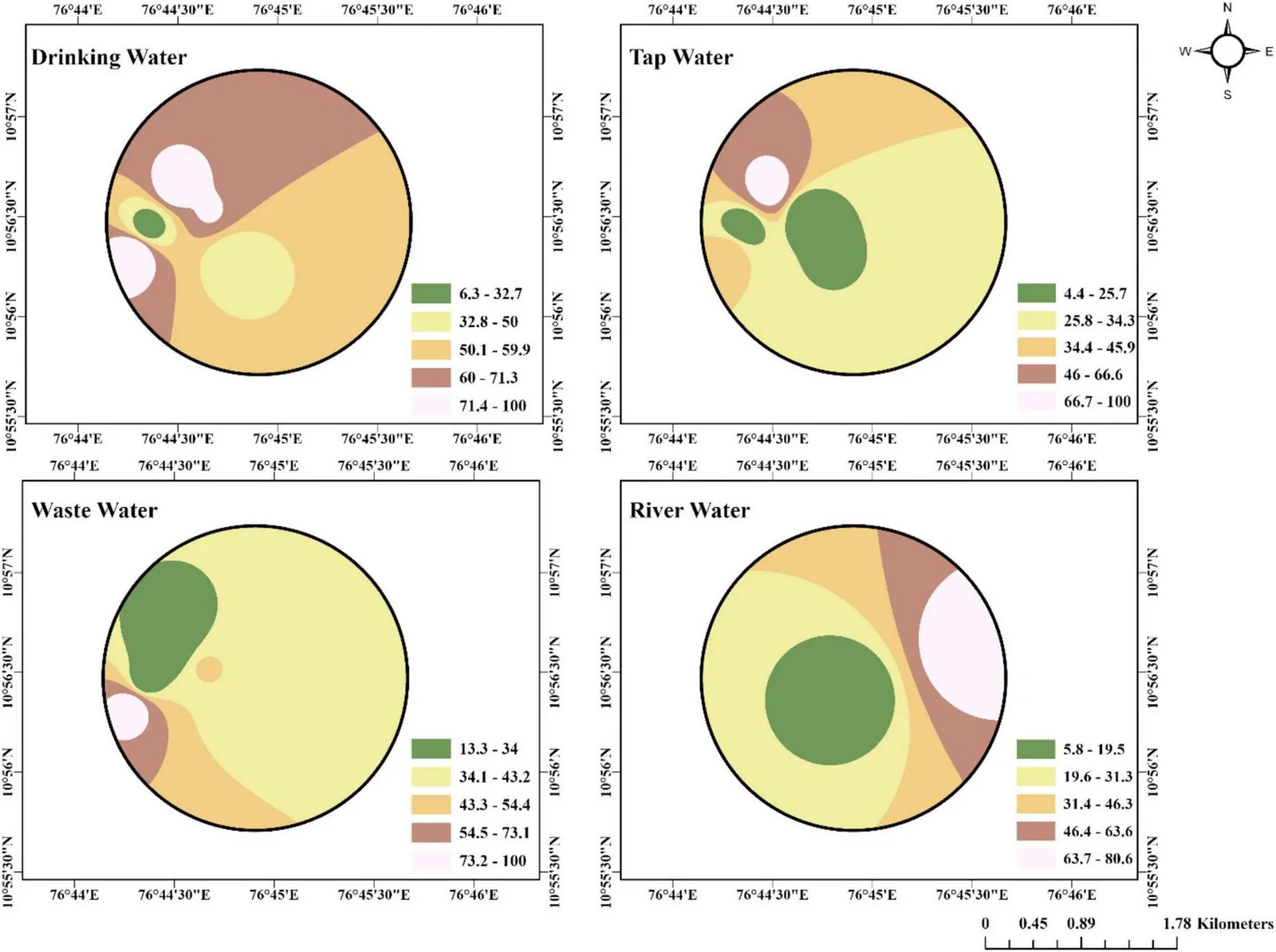
Comparative interpolated spatial distribution of relative PP composition across drinking water, tap water, wastewater, and river water systems, generated using the IDW interpolation method.

Leave-one-out cross-validation indicated substantial uncertainty for IDW interpolation, with the PE+PP fraction’s mean prediction error of 1.69, -1.08, and 4.32 percentage points and RMSE of 54.77, 53.34, and 41.46 percentage points for drinking water, tap water, and wastewater, respectively. Thus, despite low mean errors and little net directional bias overall, the comparatively high RMSE shows sufficient uncertainty that IDW surfaces should be viewed as exploratory visualizations of site-scale spatial heterogeneity rather than as definitive predictions for unsampled sites. Note: Since PP and PE+PP are complementary percentages, PP had the same RMSE, but with opposite-signed mean errors, so PP was not treated as a separate interpolation variable.

Figure 12 (PP distribution) demonstrates spatial patterns that differ from those observed for PE + PP. Compared with PE + PP, drinking water systems exhibited relatively larger areas dominated by PP, consistent with earlier results indicating a high prevalence of PP in several samples. Tap water showed moderate PP distribution with localized enrichment, reflecting site-specific variation in relative polymer composition. Wastewater also exhibited substantial variation in PP composition among sampling locations. River water exhibited strongly graded spatial patterns, characterized by alternating zones of high and low relative PP fractions; however, these patterns are based on only two sampling locations and should not be interpreted as representative of the wider river system. Combined interpolation of Figures 11, 12 indicates that relative polymer composition varied among sampling locations and water environments. However, because PE+PP and PP are mathematically complementary fractions and the interpolation uncertainty was high, these maps should not be interpreted as independent evidence of polymer-specific contamination intensity or source pathways.

The Global Moran’s I test showed no evidence that the spatial distribution of relative polymer composition was significantly autocorrelated at the global level. For drinking water, tap water, and wastewater samples, the Moran’s I coefficients were -0.782 (*p* = 0.148), -0.475 (*p* = 0.418), and -0.303 (*p* = 0.845), respectively. The same *p*-values were obtained for PP because the two fractions are complementary to each other with respect to PE+PP. Although the Moran’s I values were all negative (indicating spatial dispersion), none were statistically significantly different from a random distribution. No statistically meaningful hotspots or coldspots were detected in the sampling network when analyzed with Getis-Ord Gi* analysis. However, spatial autocorrelation and hotspot analysis were not done for river water due to the small number of sampling sites (two). Despite the observed environmental heterogeneity within sites, no statistically significant clustering or hotspots were detected in relative microplastic polymer composition. This does not eliminate the possibility of anthropogenic drivers of microplastic composition that may occur at a local scale. Rather, the present sampling density and spatial distribution limited the ability to resolve these potential drivers statistically.

Regarding the PE+PP composition, there was no statistically meaningful difference between the drinking water, tap water, wastewater, and river water (Kruskal-Wallis *H* = 1.223, *p* = 0.748). Since PP and PE+PP are complementary fractions of the reported polymer composition, the conclusion is also valid for PP. There was a statistically meaningful difference between the four groups in the turbidity results (*H* = 8.140, *p* = 0.043). However, the pairwise Mann-Whitney U comparisons with Holm correction were not important (all *p* > 0.05), which indicates that these differences are site-specific spatial trends of polymer distribution among these water systems but not statistically meaningful differences between these water categories.

The spatial distributions across the four water supplies may therefore reflect a combination of source loadings and hydrological transport processes, as well as local water distribution conditions, rather than the polymer characteristics of the different water supplies. Differences in relative polymer composition between drinking-water and tap-water locations may reflect a shift in microplastic characteristics between source and point of consumption. These results are in agreement with observations in Adediran et al. (2024) where microplastic polymer composition varied between drinking-water distribution system treatment outlets, service reservoirs and taps of consumers, and may not necessarily reflect the composition of particles upstream at treatment facilities. Accordingly, the composition of the PP- and PE-related fractions reported in the current study may be sourced from source water and/or the distribution system. Because pipe material, pipe age, water retention time, hydraulic conditions and storage terms were not included in the current study, the differences in polymer fractions between sites of tap water cannot be directly attributed to pipe abrasion or storage contamination. More frequent observations of visible plastic debris and relatively higher turbidity at the wastewater sites compared to the two river water sites may indicate larger local impacts from anthropogenic sources. However, differences in polymer profile may also be explained by mixing, dispersion, deposition and resuspension and the river would appear to be functioning as a receiving and redistribution system rather than a diluted extension of the wastewater system. Overall, polymer composition may not be utilized alone for source attribution but used in conjunction with land use, hydrological connectivity, infrastructure, and local environmental conditions.

### 3.10 Detection of antibiotic resistance gene

Results of PCR amplification of the *intI1* antibiotic-resistance-associated marker analyzed in agarose gel are shown in the Figure 13. Lane 1 is a 50 bp DNA ladder molecular size marker. Lanes 2 and 3 include the two environmental samples and amplification of the targeted *intI1* marker fragment can be confirmed by the presence of bands of approximately 280 bp. Since the size of the amplimer is within the expected range, this indicates successful amplification of the targeted marker fragment. Lane 4 is the no-template control (NTC) and, as expected, there is no amplification. Therefore, conventioanl PCR only provides qualitative presence/absence information about *intI1* in the environmental DNA, and is not able to ascertain whether the culturable bacteria in the samples contained antibiotic resistant traits, whether the *intI1* marker was present at low or high concentrations, or the level of antibiotic resistance present in the samples.

**FIGURE 13:**
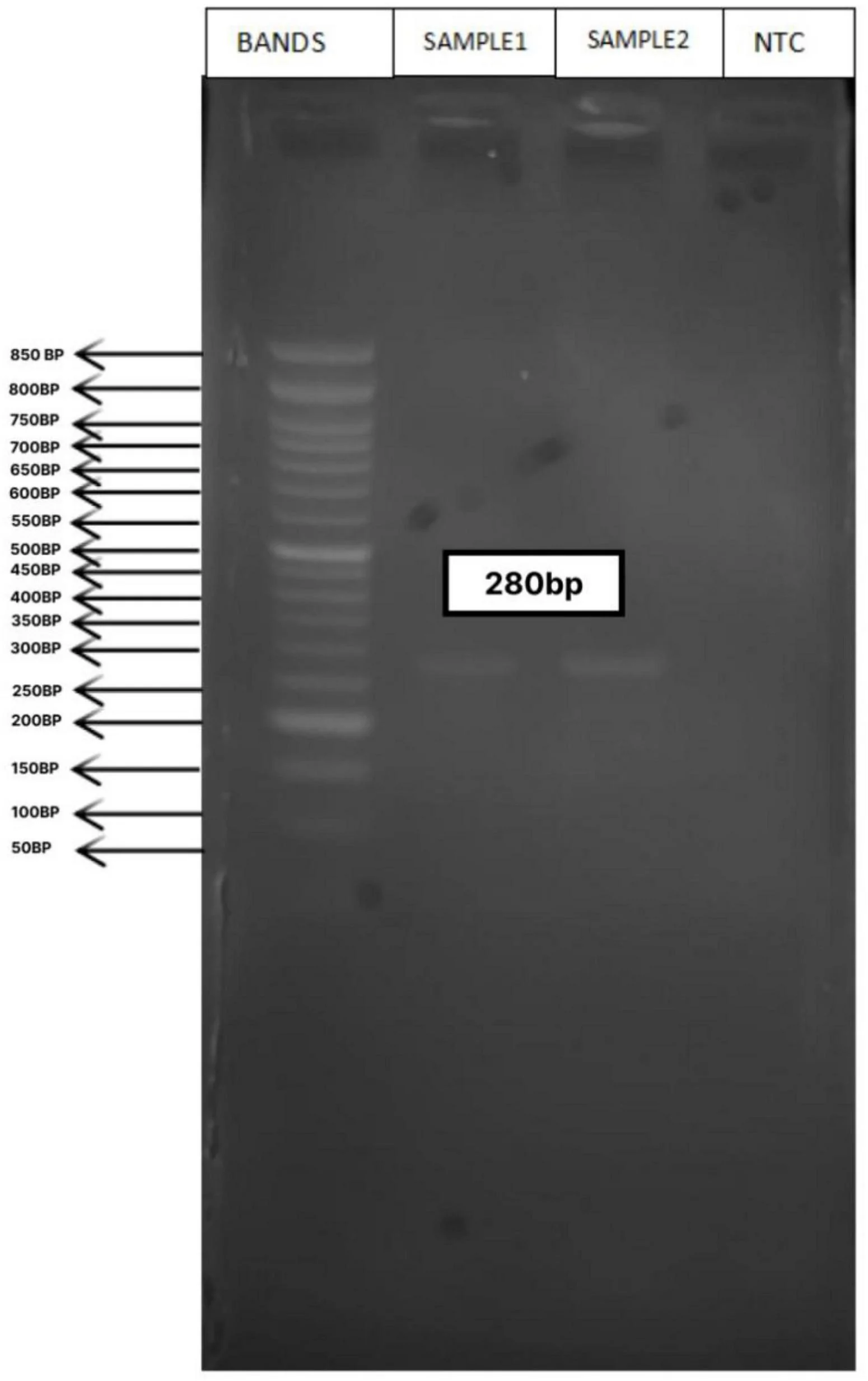
Agarose gel electrophoresis of PCR-amplified the *intI1* antibiotic-resistance-associated marker fragments showing the expected ∼280 bp bands in environmental samples alongside a 50 bp DNA ladder and a no-template control (NTC).

The detection of the *intI1* antibiotic-resistance-associated marker in the analyzed samples confirms the presence of a class 1 integron-associated marker within the studied water systems. As *intI1* is widely recognized as an indicator of anthropogenic pollution and is associated with mobile genetic elements involved in antibiotic resistance dissemination, its occurrence may indicate anthropogenic influence within the investigated environmental matrices. However, the presence of such a marker does not prove the existence of phenotypic antibiotic resistance, such antibiotic resistance determinants, or the level of antibiotic resistance. Microplastics and *intI1* have been detected in the same environmental matrices. Because of this, co-occurrence may be interpreted as associated occurrence. This does not imply that microplastics and antibiotic resistance are causally related. However, the microbial populations analyzed in this study were not specifically isolated from microplastic surfaces, and the detected *intI1* marker cannot therefore be directly attributed to microplastic-associated biofilms. Furthermore, horizontal gene transfer was not experimentally evaluated. Consequently, these findings should be interpreted as evidence of environmental co-occurrence rather than a demonstrated microplastic-mediated mechanism of antibiotic resistance dissemination.

This is consistent with more recent studies which have shown that microplastics and AR associations are very context dependent on the materials involved, the microorganisms present, and the environmental conditions experienced. Zhu et al. (2025) used a metagenomic approach to show that microplastic associated microbial communities in the aquatic environment can contain a wide diversity of AR genes, and the presence of AR genes was polymer type and bacterial community dependent. Further, Yang et al. (2025) proposed an increased risk of antibiotic resistance potentials in several microplastic biofilms based on a phenotypic-genotypic analysis of biofilms immobilized on microplastic and natural substrata in the river environment. Compared to the work of Yang et al. (2025), the current study provides less direct evidence for particle association because conventional PCR is performed on the environmental water matrix, rather than microorganisms isolated from the plastic surface. However, microplastics may not always promote antibiotic resistance. Zhai et al. (2025) report that aged microplastics were associated with antibiotic resistance genes and mobile genetic elements, and that the free radicals released from aged microplastics were negatively correlated with some antibiotic resistance-related factors in natural rivers. These conflicting results suggest that microplastic-associated AR could depend on polymer type, particle age, surface chemistry, microbial community composition, and environmental conditions. The co-occurrence of microplastics with *intI1* presented here does not conclude that microplastics promote biofilm formation and HGT but, rather, provides a rationale for future investigation of plastic-associated AR mechanistic pathways and exploration of different experimental conditions that may lead to contrasting outcomes.

### 3.11 Microbial community properties of drinking water

Gram-staining analysis of cultivable bacterial populations in drinking water samples is presented in Figures 14a–e. The micrographs indicate the presence of bacterial populations across all sampling locations despite the absence of visible turbidity, demonstrating that bacterial presence may not be reflected by visual inspection or turbidity alone. Figure 14a shows relatively sparse bacterial cells. In contrast, Figure 14b displays a comparatively greater number of stained cells with clustered formations. Figure 14c presents more uniformly distributed and densely stained cells. Figure 14d shows unevenly distributed aggregated clusters, indicating heterogeneity in the microscopic distribution of bacterial cells. Figure 14e indicates a moderate microbial presence, characterized by dispersed but clearly identifiable bacterial cells.

**FIGURE 14:**
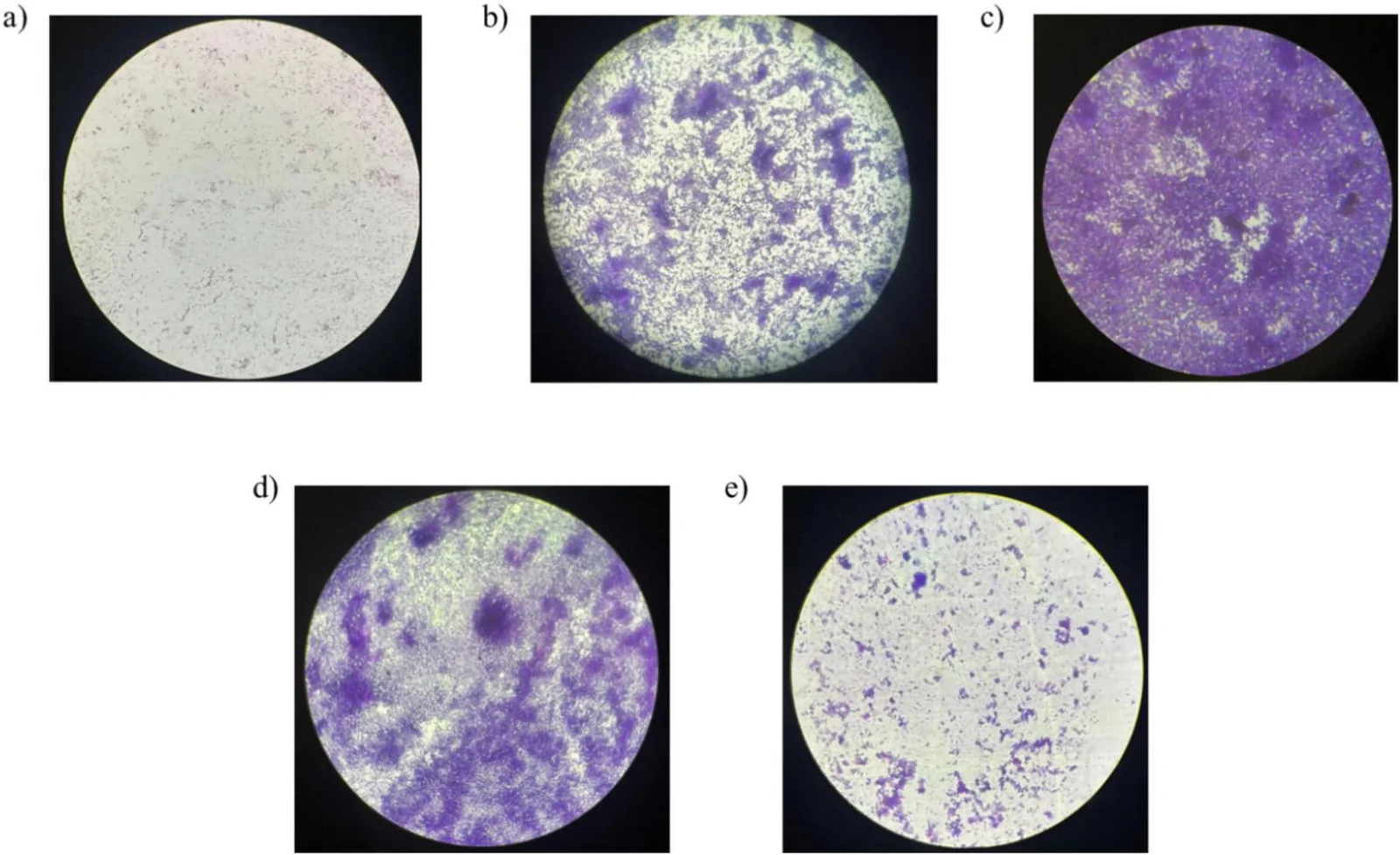
Gram-stained microscopic images of cultivable bacterial populations observed in drinking water samples: **(a)** Sample 1, **(b)** Sample 2, **(c)** Sample 3, **(d)** Sample 4, and **(e)** Sample 5, illustrating qualitative variation in bacterial cell distribution across sampling locations.

The presence of purple-stained regions in several samples indicates the presence of Gram-positive bacteria, whereas lightly stained or pink-stained regions suggest the occurrence of Gram-negative bacteria, confirming the coexistence of different bacterial Gram reactions within the drinking water system. Variations in the observed cell distribution among samples indicate site-specific differences in the cultivable bacterial populations; however, the present Gram-staining analysis does not provide quantitative bacterial abundance, species-level identification, or comprehensive microbial community profiling. Notably, the detection of bacterial populations in all samples, including visually clear water, indicates that drinking water systems can contain cultivable bacteria even under low-turbidity conditions. Although microplastics can provide attachment surfaces for microorganisms as reported in previous studies, bacterial attachment to the microplastic particles was not directly examined in the present study. Therefore, these observations should not be interpreted as evidence of microplastic-associated biofilm formation, enhanced microbial survival, or horizontal gene transfer.

### 3.12 Microbial community properties of tap water

Figures 15a–e presents Gram-stained microscopic images of cultivable bacterial populations observed in tap water samples. Presence of both Gram positive (purple/violet) and Gram negative (pink/red) bacterial population in the micrographs suggests the coexistence of both types of bacterial Gram reactions in the environment of the tap water samples. Figure 15a has a greater ratio of pink cells indicating Gram-negative bacteria and the Figure 15b has more purple cells indicating Gram-positive bacteria.

**FIGURE 15:**
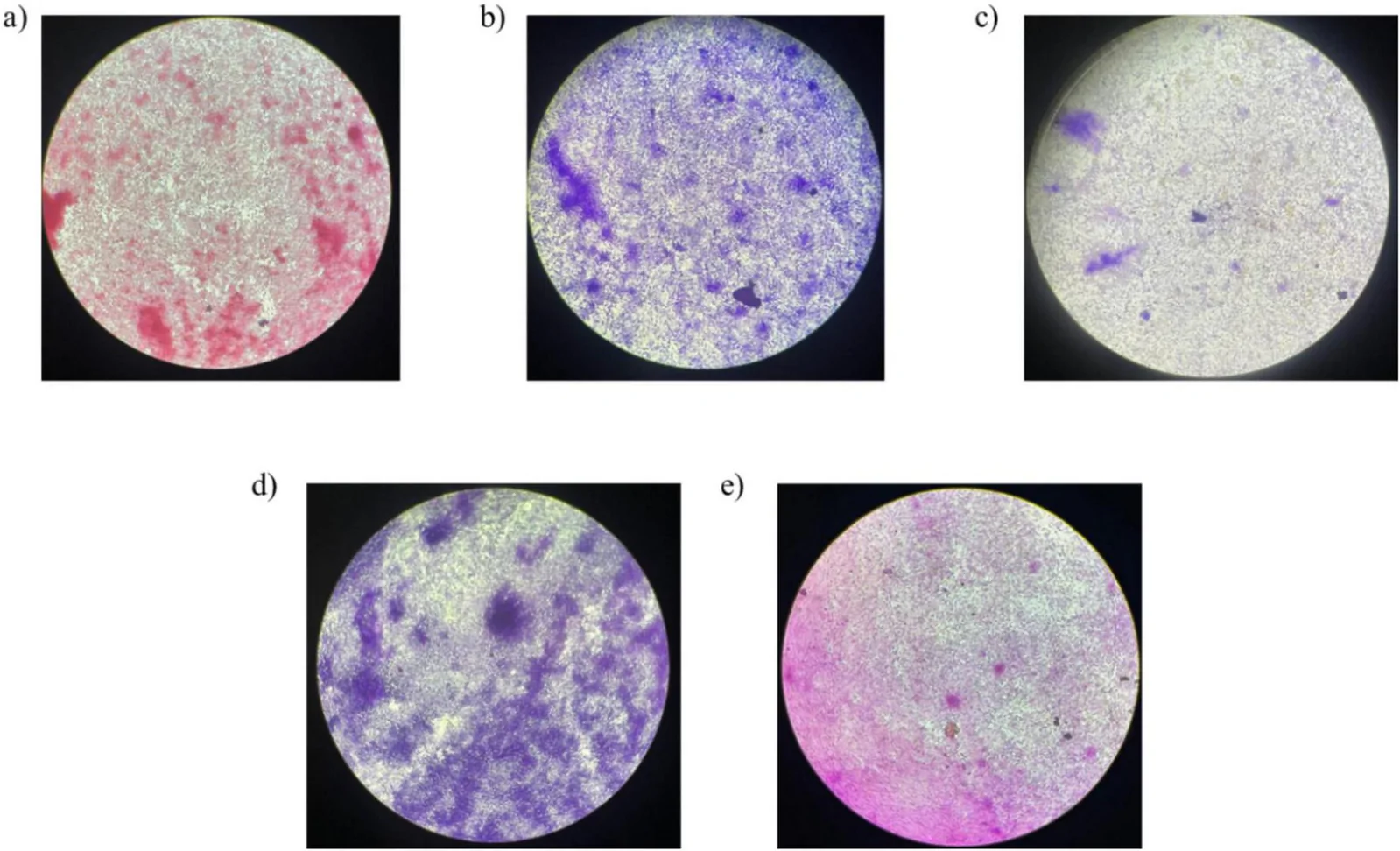
Gram-stained microscopy of the populations of cultivable bacteria in the tap water samples: **(a)** Sample 1, **(b)** Sample 2, **(c)** Sample 3, **(d)** Sample 4, and **(e)** Sample 5. Showing the qualitative variation of the proportion of Gram-positive (purple/violet) to Gram-negative (pink/red) bacteria in the samples from the different sampling sites.

The bacteria were not abundant and in Figure 15c there are less signs of clustering. In Figure 15d there are clusters of purple-stained cells, indicating the presence of bacteria in aggregations. Figure 15e shows a mixed population of pink (Gram negative) and purple (Gram positive) stained cells, indicating the presence of both types of bacteria. The observed variations in cell distribution and staining characteristics among samples demonstrate site-specific heterogeneity in the cultivable bacterial populations. Although Gram-negative bacteria could potentially harbor mobile genetic elements (e.g., class 1 integrons), this was not possible based on the Gram-staining to ascertain whether *intI1* was present in specific Gram-negative groups or link them to our PCR results. Gram-positive bacteria have also been noted as part of our culturable community, although their role, if any, in surface colonization or biofilm formation remains to be elucidated. These results, together with the fact that culturable bacteria could be identified in all samples, including those with low turbidity, indicate that tap water systems may contain bacteria even in the absence of visible contamination (Afonso et al., 2025). Although microplastics can provide attachment surfaces for microorganisms, bacterial attachment to the isolated microplastic particles was not directly examined in the present study. Therefore, these observations should not be interpreted as evidence that microplastics enhanced microbial persistence, biofilm formation, or horizontal gene transfer.

### 3.13 Microbial community properties of wastewater

Figures 16a–e presents Gram-stained microscopic images of cultivable bacterial populations observed in wastewater samples. The micrographs demonstrate considerable bacterial presence and qualitative heterogeneity across all sampling locations, with the presence of both Gram-negative (pink/red) and Gram-positive (purple/violet) bacterial populations. The dense clusters of pink-stained cells observed in Figure 16a indicate a comparatively greater presence of Gram-negative bacteria. The mixed staining pattern of cells as given in Figure 16b, at this level of magnification suggests the presence of both Gram-positive and Gram-negative bacteria. Regular clusters of purple-stained cells indicate the presence of Gram-positive bacteria as presented in Figure 16c, while non-clustered and scattered microbial cells as shown in Figure 16d indicate the presence of a heterogeneous distribution of bacterial cells. In contrast, the dense and relatively homogeneous microbial lawn can be seen to contain a mixture of the Gram-positive and Gram-negative bacteria in Figure 16e.

**FIGURE 16:**
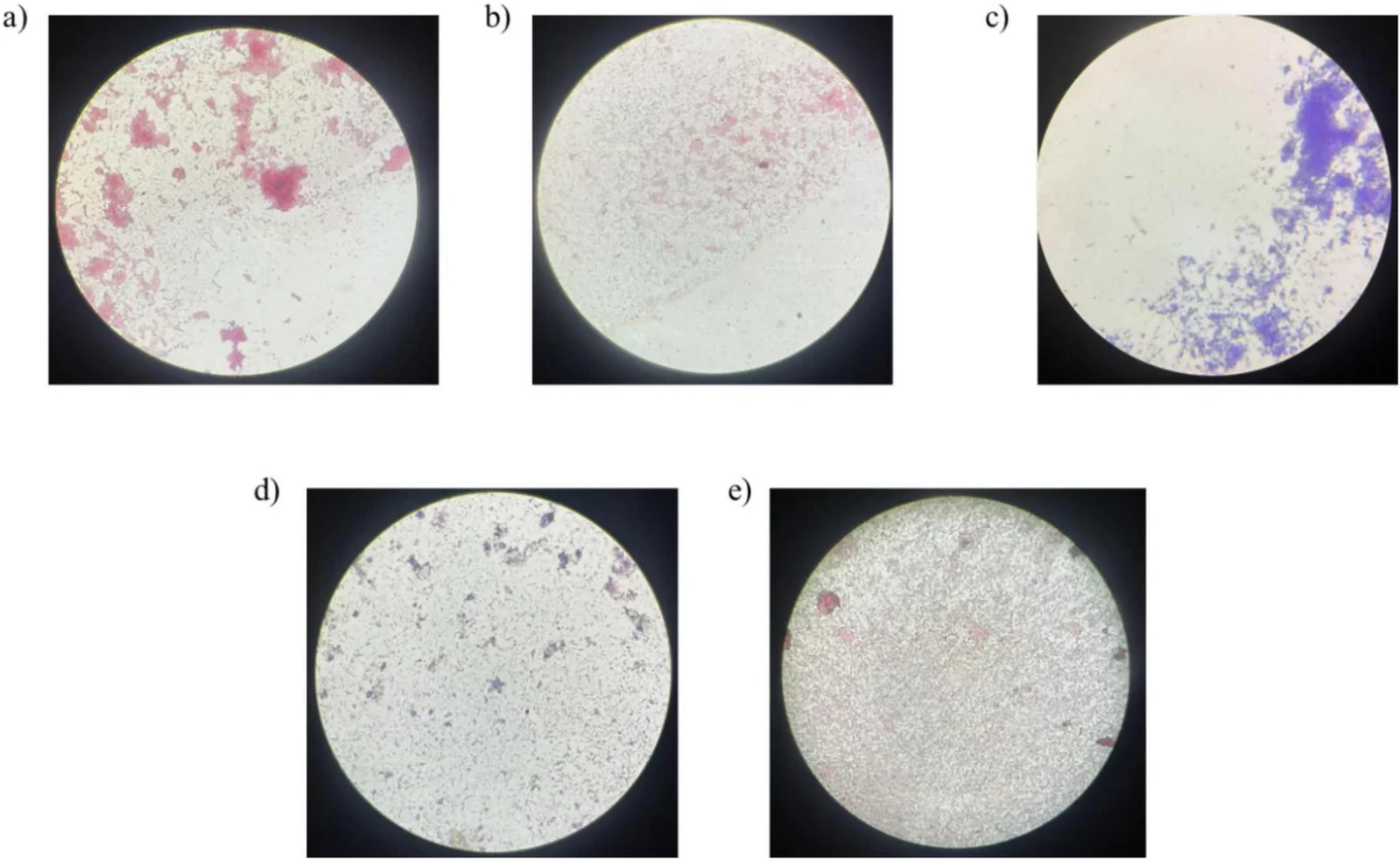
Microscopic images of the Gram-stained culturable bacterial communities identified in the wastewater samples. **(a)** Sample 1; **(b)** Sample 2; **(c)** Sample 3; **(d)** Sample 4; and **(e)** Sample 5. Sampled sites observed a difference in bacterial spread and the presence of Gram-positive (purple/violet) and Gram-negative (pink/red) bacteria.

Bacterial communities were rich and diverse in all wastewater samples analyzed in this study. There were also changes in Gram-negative populations. In several samples, Gram-negative bacteria were visually prominent; however, Gram staining alone cannot determine whether these organisms carried the *intI1* marker or other antibiotic resistance-associated determinants detected by PCR. In addition, the presence of dense microbial clusters and aggregated cellular structures indicates bacterial aggregation but does not by itself confirm biofilm formation or horizontal gene transfer. Compared with drinking and tap water systems, wastewater samples showed more visually pronounced bacterial cell aggregation in the examined micrographs; however, the present culture-based and Gram-staining approach does not permit quantitative comparison of microbial abundance or diversity among the different water systems (La Rosa et al., 2025). The coexistence of microplastics, cultivable bacterial populations, and the *intI1* marker within the wastewater environment demonstrates environmental co-occurrence; however, bacterial attachment to microplastic surfaces and microplastic-mediated dissemination of antibiotic resistance were not directly evaluated in the present study.

## 4 Conclusion

This study demonstrates that microplastic pollution in rural water systems is detectable across the investigated water environments but exhibits substantial site-specific variability in relative polymer composition. It also confirms the ineffectiveness of visual methods and customary physicochemical parameters in quantifying microplastics in water samples and confirms its presence and relative polymer profile in the tested water samples. Wastewater systems, in particular, were associated with high turbidity (14.3 NTU) and inter-sample variation of polyolefins (with PE+PP fractions up to 86.79%). The fraction of PE+PP in tap water also varied site-specifically and reached values as high as 95.73%. However, the contributions of pipelines, storage tanks and distribution networks to tap water systems could not be separated in the present study. The different polymer composition between the two rivers may lead to some differences in the spatial patterns, but the lack of replicates impedes a more general assessment. Polyolefin signatures were seen in the drinking water sample, even at zero-turbidity, with polypropylene making up to 100% of relative polymer composition at some locations.

The PE+PP composition among drinking water, tap water, wastewater, and river water did not considerably differ (Kruskal-Wallis *H* = 1.223, *p* = 0.748). However, the turbidity levels between the four types of water did differ (Kruskal-Wallis *H* = 8.140, *p* = 0.043). However, pairwise comparisons did not remain significant after Holm correction. Global Moran’s I and Getis-Ord Gi* analyses further showed no statistically significant spatial autocorrelation, hotspots, or coldspots in relative polymer composition. In addition, the relatively high RMSE values obtained during IDW cross-validation indicated considerable interpolation uncertainty. Therefore, the GIS outputs should be interpreted as exploratory representations of site-scale spatial heterogeneity rather than definitive evidence of contamination hotspots or spatially continuous concentration gradients.

Microbial analysis confirmed the presence of cultivable Gram-positive and Gram-negative bacterial populations across the investigated water sources, including drinking water. Detection of the *intI1* antibiotic-resistance-associated marker further demonstrated the presence of a class 1 integron-associated marker linked with anthropogenic contamination and mobile genetic elements associated with antibiotic resistance. However, conventional PCR was used only for qualitative detection and therefore does not provide information on *intI1* abundance or gene load. It is also important to note, however, that co-occurrence with microplastics and the *intI1* antibiotic-resistance-associated marker does not imply a causal link between the two, or that a mechanistic link exists. Microorganisms were not specifically isolated from microplastic surfaces, and microplastic-associated biofilm formation and horizontal gene transfer were not experimentally evaluated. Therefore, the present findings do not establish that microplastics facilitated microbial colonization, gene exchange, or antibiotic resistance dissemination. In the context of SDGs, the findings have potential relevance to SDG 6 by demonstrating the occurrence of microplastics in rural water systems, SDG 3 through the detection of an antibiotic resistance-associated environmental marker, and SDG 13 by illustrating the broader need to understand emerging contaminants under changing environmental conditions.

The results further indicate that rural environments require greater attention in emerging-contaminant monitoring rather than being considered confirmed hotspots on the basis of the present dataset. A key limitation of existing water quality monitoring frameworks is the limited integration of microplastic assessment and microbial risk evaluation. Therefore, future monitoring and research should combine standardized quantitative microplastic measurements, including particle abundance per unit volume and systematic characterization of particle size and morphology, with quantitative molecular approaches such as qPCR, 16S rRNA sequencing, and metagenomic analysis. Direct characterization of microplastic-associated biofilms and resistance determinants would also be required to establish mechanistic relationships. Expanded spatial sampling together with repeated seasonal and interannual monitoring would improve assessment of temporal variability and spatial representativeness. FTIR spectroscopy, molecular diagnostics, and GIS-based spatial analysis can provide complementary information within such integrated monitoring frameworks. The present study should therefore be regarded as an exploratory village-scale assessment providing baseline evidence of microplastics, cultivable bacterial populations, and the *intI1* marker in rural water environments, while highlighting the need for larger and more mechanistically resolved investigations.

## Data Availability

The original contributions presented in the study are included in the article/supplementary material, further inquiries can be directed to the corresponding authors.
